# SGCD: High‐Resolution Spatial Domain Characterization via Data Interpolation and Cell‐Type Deconvolution

**DOI:** 10.1002/advs.202506176

**Published:** 2025-06-20

**Authors:** Tianjiao Zhang, Shenghe Li, Ruolan Zhang, Hongfei Zhang, Zhongqian Zhao, Hao Sun, Zhenao Wu, Guohua Wang

**Affiliations:** ^1^ College of Computer and Control Engineering Northeast Forestry University No. 26 Hexing Road, Xiangfang District Harbin 150040 China; ^2^ Faculty of Computing Harbin Institute of Technology No. 26 Hexing Road, Xiangfang District Harbin 150001 China

**Keywords:** cell type deconvolution, data interpolation, spatial domain recognitions, spatial transcriptomics

## Abstract

The rapid advancement of spatial transcriptomics has provided a critical data foundation for the high‐resolution characterization of tissue spatial domains. Traditional methods for spatial domain identification primarily rely on gene expression data from sampled spots in low‐resolution spatial transcriptomic data, often overlooking valuable information between spots that can be crucial for domain identification. Furthermore, these methods are limited by their focus on gene expression data from neighboring spots, without fully integrating prior knowledge of cell types within the tissue's spatial structure. To address these challenges, SGCD, a novel method for tissue spatial domain identification based on data interpolation and cell type deconvolution is proposed. SGCD utilizes interpolation techniques to estimate gene expression data for cells in the gaps between spots and applies deconvolution to extract cell type information from both spots and interstitial regions. By integrating gene expression, cell type, and spatial location data, SGCD achieves accurate delineation of complex spatial domains through graph contrastive learning. Evaluations on various publicly available datasets, including the human dorsolateral prefrontal cortex, mouse brain, pancreatic ductal adenocarcinoma, and breast cancer, demonstrate that SGCD significantly outperforms existing methods in both accuracy and detail, offering strong support for advancing the understanding of tissue functions and disease mechanisms.

## Graphical abstract

SGCD presents a novel approach for tissue spatial domain identification by employing interpolation to estimate inter‐spot gene expression and deconvolution to resolve cell‐type composition in both sampled and interstitial regions. By integrating gene expression, cell type, and spatial coordinates within a graph contrastive learning framework, SGCD achieves enhanced accuracy and resolution, advancing insights into tissue function and disease mechanisms.

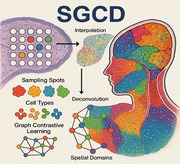

## 1 Introduction

In recent years, spatial transcriptomics technology has advanced rapidly, not only systematically characterizing cellular heterogeneity while preserving the tissue spatial context,^[^ ^1^, ^2^, ^3^, ^4^, ^5^, ^6^ ^]^ but also providing powerful tools for the precise delineation of spatial domains within tissues, thereby deepening our understanding of cell‐environment interaction mechanisms. The available spatially resolved transcriptomic techniques have two major categories:^[^ ^7^ ^]^ i) in situ hybridization or in situ sequencing methods, such as seqFISH,^[^ ^8^ ^]^ MERFISH,^[^ ^7^ ^]^STARmap,^[^ ^3^ ^]^ and FISSEQ.^[^ ^9^ ^]^ These methods enable cellular or even subcellular resolution by directly detecting predefined RNA targets in situ, but their multiplexing capacity is limited, typically allowing the detection of only a few hundred to a few thousand genes; ii) in situ capture techniques, such as spatial transcriptomics (ST),^[^ ^4^ ^]^ SLIDE‐seq,^[^ ^2^ ^]^ ZipSeq,^[^ ^10^ ^]^ and HDST.^[^ ^11^ ^]^ These methods capture transcripts in situ within tissues and then perform ex vivo sequencing, enabling unbiased analysis of the entire transcriptome. However, the “point‐based” capture strategy has inherent limitations. For instance, platforms like 10X Visium feature 4992 spots on a slide, each with a diameter of 55 µm and arranged with an ≈100 µm center‐to‐center distance between adjacent spots. This configuration leaves ≈70% of the region's gene expression data unmeasured (calculated as $\left(1-\frac{4992\;\times \;\pi {\left(\frac{55}{2}\right)}^{2}}{6500^{2}}\right)$) resulting in a significant information gap. Traditional spatial domain identification methods primarily rely on the spatial proximity hypothesis—that is, they delineate spatial domains by comparing the overall expression patterns between adjacent spots. Non‐spatial clustering methods (such as K‐means,^[^ ^12^ ^]^ Louvain,^[^ ^13^ ^]^ Seurat^[^ ^14^ ^]^) often fail to capture tissue continuity and, therefore, are frequently unable to fully reflect the actual spatial patterns of the tissue. These methods rely solely on gene expression data and overlook the spatial relationships between spots, making it difficult to effectively identify the inherent structural features of the tissue. To address this limitation, several improved methods that incorporate spatial information have been proposed in recent years. For example, Giotto^[^ ^15^ ^]^ employs a hidden Markov random field (HMRF) model to detect and identify spatial domains with coherent gene expression patterns by fully utilizing spatial dependencies between spots; BayesSpace^[^ ^16^ ^]^ uses Bayesian statistical methods to integrate spatial neighborhood information for optimized clustering results; STAGATE^[^ ^17^ ^]^ and GAADE^[^ ^18^ ^]^ combine Graph Attention Auto‐Encoder frameworks to further integrate spatial information with gene expression profiles. Furthermore, studies have shown that tissue histopathology images can effectively predict gene expression,^[^ ^19^ ^]^ leading to methods (such as SpaGCN^[^ ^20^ ^]^ and stLearn^[^ ^21^ ^]^) that improve spatial domain identification by integrating neighborhood information and morphological features, while DeepST^[^ ^22^ ^]^ takes it a step further by combining image features, gene expression, and spatial localization.

Despite the significant progress made by these methods, they still face challenges in fully exploiting the spatial relationship and topological features between cells or spots in the unlabeled data. To address this, some methods have introduced contrastive learning, which leverages the intrinsic structure and properties of the data to learn low‐dimensional representations, thereby alleviating the challenges posed by limited labeled data. For example, conST^[^ ^23^ ^]^ integrates multimodal data at the spot, subcluster, and global levels for contrastive learning; ConGI^[^ ^24^ ^]^ designs a joint learning strategy that includes gene expression, images, and a contrastive loss function between them; GraphST^[^ ^25^ ^]^ integrates spatial information and gene expression through graph self‐supervised contrastive learning; MuCoST^[^ ^26^ ^]^ enhances spot dependencies by combining gene expression correlations and spatial proximity; and recently, STAIG^[^ ^27^ ^]^ combines graph contrastive learning with high‐performance feature extraction to integrate gene expression, spatial coordinates, and histological images. However, despite these breakthroughs in combining spatial information, histological images, and gene expression data, these methods still primarily rely on the spatial proximity hypothesis, assuming that adjacent spots share similar gene expression patterns. While this hypothesis reveals certain aspects of the spatial distribution of gene expression and the regional characteristics of cell types, it does not fully exploit the cellular composition information within gene expression data. As a result, these methods may fail to accurately capture the spatial distribution and functional characteristics of cell populations driven by specific biological processes, thus limiting the precise characterization of spatial domain details and biological functions.

To address the limitations of the aforementioned methods, we propose a novel tissue spatial domain identification method based on data interpolation and cell type deconvolution—SGCD. SGCD addresses the issue in traditional methods where low‐resolution spatial transcriptomics (e.g., 10X Visium) cannot utilize the interstitial information between adjacent spots by introducing a reasonable interpolation approach to fill in the missing gene expression data and improve the continuity of data acquisition. To tackle the problem of insufficient use of cellular composition information in traditional methods, SGCD integrates the interpolated data for deconvolution, fully extracting the cellular composition information of each spot and combining it with spatial location data to construct a weighted adjacency matrix. Furthermore, by dynamically adjusting the balance between spatial proximity and cell type similarity through the parameter *γ*, SGCD models the dual dependencies of both factors. We systematically evaluated SGCD on multiple publicly available datasets, including the human dorsolateral prefrontal cortex, mouse brain, human pancreatic ductal adenocarcinoma, and breast cancer. Experimental results demonstrate that SGCD significantly outperforms existing mainstream methods in both spatial domain recognition accuracy and detailed characterization, providing solid data support and technical assurance for in‐depth analysis of tissue functions and disease mechanisms.

## 2 Result

### 2.1 Overview of SGCD

**Figure 1:**
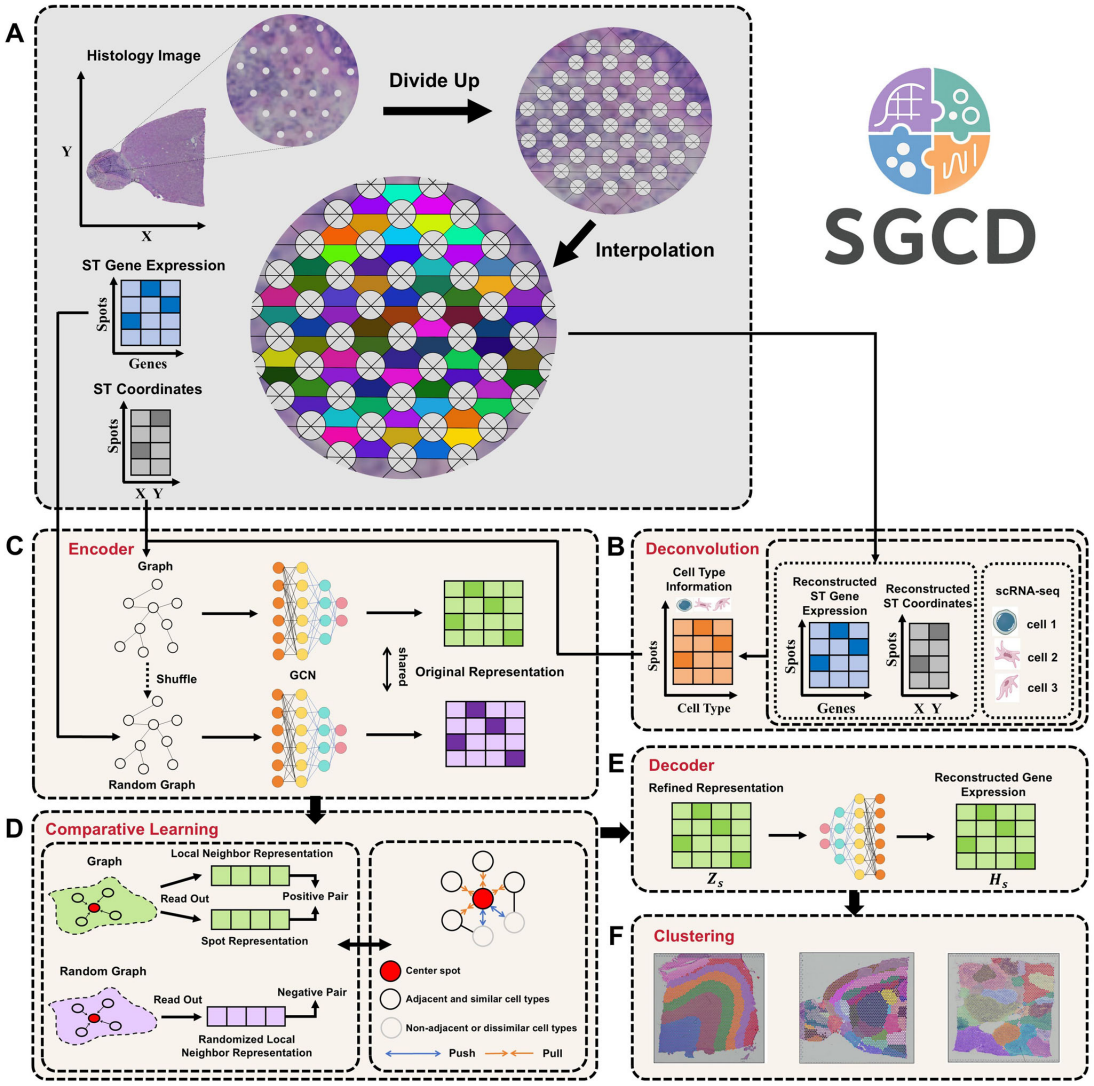
Overview of the SGCD Framework Workflow. A) Interpolation Process for Missing Data Between Points: First, the spatial transcriptomic data is divided based on a structured arrangement, and the gaps between the points are evenly partitioned into regular shapes. The centroids of these shapes are then assigned spatial coordinates. Next, the SpaVAE method is used to learn the latent relationship between the original gene expression data and spatial coordinates, further interpolating the gene expression data for the gap regions. B) Deconvolution Process Combining Single‐Cell RNA Sequencing Data with Spatial Transcriptomic Data: The original spatial transcriptomic data is concatenated with the newly interpolated data to form a reconstructed spatial transcriptomic dataset. This dataset is then combined with single‐cell RNA sequencing data for deconvolution to extract more detailed cellular composition information. C) Weighted Graph Construction Based on Cell Type Similarity and Spatial Coordinates: A weighted graph structure is constructed using information about cell type similarity and spatial coordinates. Random graphs are generated by applying perturbations, and a GCN encoder is utilized to extract node features. D) Self‐Supervised Contrastive Learning for Contextual Node Information: A self‐supervised contrastive learning module generates positive and negative sample pairs (i.e., the original image and the perturbed random graph), which helps capture the local contextual information of nodes. E) Reconstruction of Latent Representations via the Decoder: The decoder is employed to reconstruct the learned latent representation. F) Spatial Domain Recognition Results Using SGCD on Different Spatial Transcriptomics Datasets: The final results demonstrate the spatial domain recognition capabilities of SGCD when applied to various spatial transcriptomics datasets.

SGCD is a graph‐based self‐supervised contrastive learning framework designed to generate robust spatial transcriptomics low‐dimensional representations by integrating gene expression, spatial information, and cell type information. As shown in **Figure** **1**A, the process begins with a rational division of the spatial transcriptomics data based on a regular arrangement. In this study, we primarily use data from the 10X Visium platform, where the sampling points are arranged in a regular hexagonal pattern. Therefore, the tissue section is divided row by row along the horizontal direction, and within each row, it is further subdivided along the left and right 45‐degree diagonals, thereby defining a complete gap region between three points. Subsequently, based on the original spatial coordinate data, the centroids of the divided regions are taken as the new spatial coordinates for the gap areas. Next, the SpaVAE^[^ ^28^ ^]^ method is used to learn the latent relationship between gene expression and spatial coordinates in the original spatial transcriptomics data, and gene expression data is interpolated for the divided gap regions. This process not only ensures a reasonable division and filling of the gap areas between the original discrete points but also reconstructs the spatial relationships between sampling points continuously. After these steps, methods like Spatialprompt^[^ ^29^, ^30^, ^31^ ^]^ are employed to combine the reconstructed spatial transcriptomics data with single‐cell RNA sequencing data for deconvolution, providing richer cell‐type composition information (Figure 1B). The cell type information obtained from deconvolution is then used to calculate the Jensen‐Shannon Divergence (JSD) similarity, and a weighted graph structure is constructed by combining spatial coordinate information. The weight of spatial proximity and cell type similarity is dynamically adjusted using the parameter *γ*, modeling the dual dependence between them. Then, a Graph Convolutional Network (GCN) encoder is used to extract features from the gene expression data and the weighted graph (Figure 1C), further refining node embeddings under the self‐supervised contrastive learning module. Specifically, SGCD generates “random graphs” by randomly shuffling gene expression vectors, creating a graph with the same topology as the original but with perturbed features. This process generates positive and negative sample pairs for each node (Figure 1D). The local context information of each node is then aggregated from the embeddings of its neighboring nodes and passed through a nonlinear transformation (such as a sigmoid function). This leads to a strong contrast between the real context in the original graph and the false context in the random graph, encouraging the model to learn similar embeddings for spatially adjacent nodes with similar cell types, and significantly different representations for nodes that are either not adjacent or have a large cell type discrepancy. Moreover, SGCD defines contrastive losses on both the original and random graphs, combining them to form symmetric contrastive losses that enhance training stability. Finally, a decoder is used to map the learned low‐dimensional representations back to the original space (Figure 1E), reconstructing the gene expression matrix and jointly optimizing it with the contrastive loss to ensure the retention of key gene expression features while capturing richer spatial context. Figure 1F shows the spatial domain division results of SGCD on real spatial transcriptomics data. In this study, the interpolation method SpaVAE^[^ ^28^ ^]^ and the deconvolution method Spatialprompt^[^ ^29^, ^30^, ^31^ ^]^ were applied using the default parameter settings recommended in their original publications.

### 2.2 SGCD Aids in the Precise Analysis of the Spatial Hierarchical Structure of Tissues

**Figure 2:**
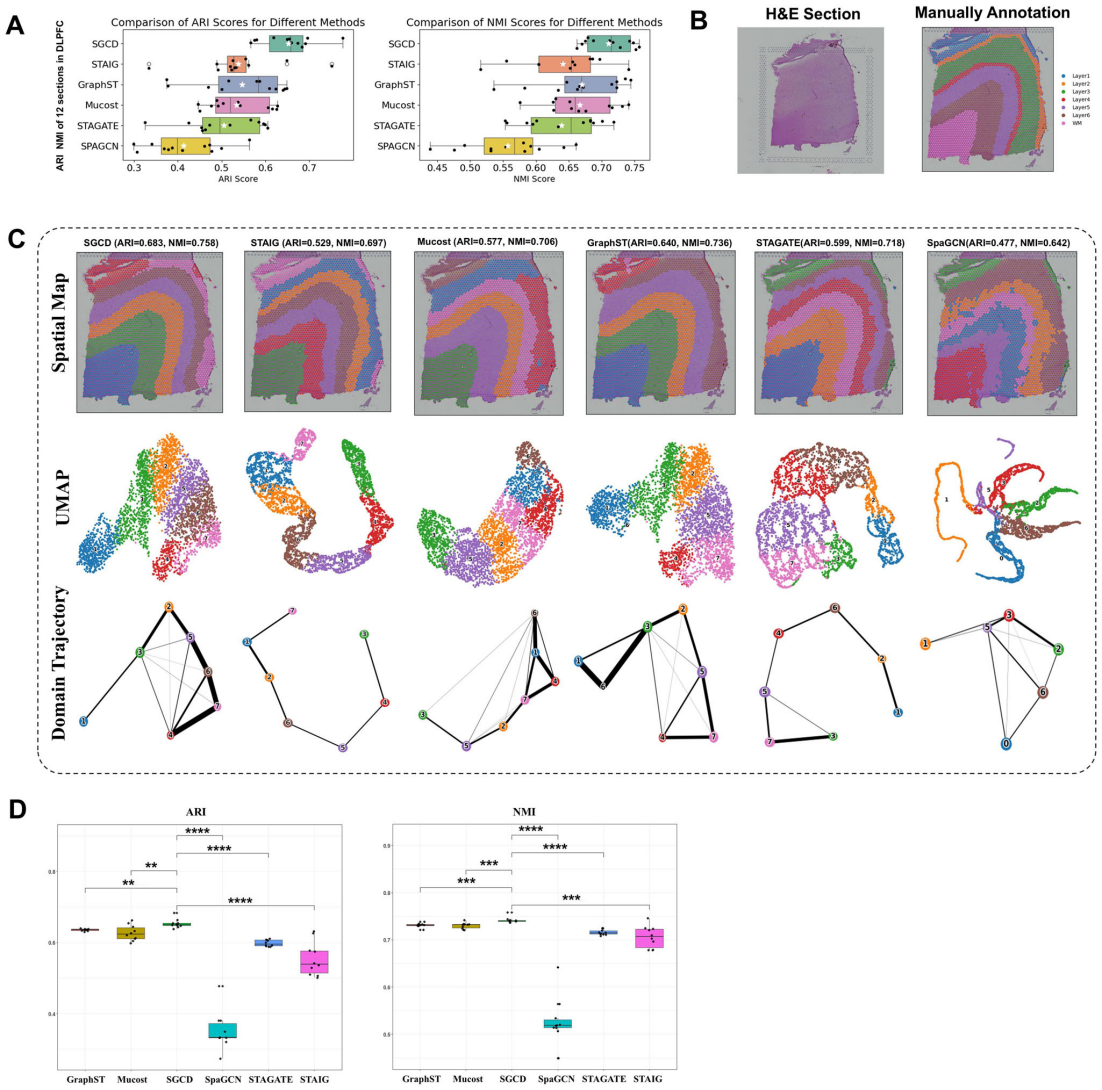
Application of SGCD on the DLPFC dataset. A) Box plots of clustering evaluation metrics (ARI and NMI) across 12 slices of the DLPFC dataset, comparing SGCD with five baseline methods. B) Histological image and manual annotations of slice #151673. C) Spatial domain identification, UMAP, and PAGA plots for slice #151673 using SGCD and five other methods. D) T‐test results assessing the statistical significance of clustering differences between SGCD and other methods.

SGCD enables precise analysis of the spatial structure of the DLPFC tissue. We first evaluated the representation learning performance of SGCD on benchmark datasets. The 10X Visium dataset of the human dorsolateral prefrontal cortex (DLPFC) is a widely used benchmark for evaluating spatial clustering performance. This dataset consists of 12 slices, each containing 4 or 6 DLPFC layers and one white matter (WM) layer. These structural domains have been annotated using morphological features and marker genes,^[^ ^32^ ^]^ providing valuable resources to assess the clustering accuracy of the learned representations (**Figure** **2**B). We compared SGCD with STAIG, MuCoST, GraphST, STAGATE, and SpaGCN on the spatial domain accuracy of all 12 slices in the DLPFC dataset (Figure 2A), using the Adjusted Rand Index (ARI) and Normalized Mutual Information (NMI). SGCD achieved the best performance, with the highest average ARI and NMI scores of 0.651 and 0.710, respectively. GraphST achieved average ARI and NMI scores of 0.547 and 0.670, respectively. STAIG's average ARI and NMI scores were 0.538 and 0.641, respectively. MuCoST's average ARI and NMI scores were 0.534 and 0.667, respectively. STAGATE's average ARI and NMI scores were 0.505 and 0.639, respectively. SpaGCN's average ARI and NMI scores were 0.413 and 0.6557 (Figure 2A). Detailed results for all 12 slices of the DLPFC dataset are provided in Figures S1 and S3 (Supporting Information).

To showcase the detailed performance of SGCD, we provide the spatial domain identification results for slice #151673, which includes six DLPFC layers and one WM layer (Figure 2C–F). First, SGCD demonstrated the strongest clustering performance, achieving high scores of ARI = 0.683 and NMI = 0.758 (Figure 2C). Its spatial domain identification results clearly delineated the boundaries between the six DLPFC layers and the WM layer, providing a solid foundation for subsequent spatial data analysis. Additionally, the UMAP plot shows a compact and well‐separated distribution of data clusters (Figure 2C), while the PAGA trajectory plot clearly illustrates the smooth transition from Layer 1 to Layer 6 and then to the WM layer (Figure 2C, bottom). The statistical results of the t‐test show that SGCD significantly outperforms other methods, with differences reaching significance levels of p < 0.05 (^*^), p < 0.01 (^**^), and p < 0.001 (^***^) (Figure 2D). These results firmly demonstrate its advantages in clustering accuracy and hierarchical reasoning. STAIG also showed some ability in clustering and trajectory inference, scoring ARI = 0.529 and NMI = 0.697. Its spatial domain identification results were able to reveal the spatial evolution of the data from Layer 1 to Layer 6 and to WM. Although overall performance was not as strong as SGCD, the UMAP plot showed relatively distinct cluster distributions, and it still had reference value in capturing spatial structure changes. MuCoST achieved clustering performance with ARI = 0.577 and NMI = 0.706, showing good overall performance. However, there were some limitations in the boundary separation between certain layers (e.g., between Layer 2 and Layer 4), which resulted in unclear transitions between layers in the PAGA trajectory plot. This blurry boundary somewhat affected the precise characterization of the data's spatial evolution. GraphST also showed high clustering accuracy (ARI = 0.640, NMI = 0.736), effectively partitioning different spatial domains. However, its boundary recognition between some layers was slightly lacking, leading to trajectory inference in the PAGA plot that was not as clear as SGCD's. Overall, GraphST captured the general hierarchical structure, but it was less refined than SGCD in terms of details. In comparison, STAGATE performed relatively poorly. The boundaries between different spatial domains in its clustering results were unclear, and the distribution of data points in the UMAP plot was mixed, failing to show clear separation. Due to unclear clustering, the PAGA trajectory inference did not present a reasonable hierarchical structure, making the spatial evolution analysis less accurate. SpaGCN's clustering results were the most chaotic, with both ARI and NMI scores being low, and there was no clear separation between different layers in its spatial domain identification process. The UMAP plot showed significant overlap between clusters, and the PAGA trajectory plot exhibited clear fuzziness and confusion, failing to reveal the true hierarchical evolution. This highlights SpaGCN's significant shortcomings in accurately distinguishing and characterizing spatial structure. Overall, the analysis of each method reveals that SGCD leads in clustering accuracy, representation learning, and hierarchical reasoning, offering the clearest and most precise spatial domain identification, while other methods exhibit varying degrees of boundary fuzziness or unclear transitions between layers.

To address the gap issue in 10X Visium data sampling points, SGCD employs a unique preprocessing and modeling pipeline: it enhances spatial continuity through interpolation, extracts precise cell type information via deconvolution, and constructs a weighted adjacency graph based on spatial coordinates to capture interactions between spatial neighborhoods and cellular features. This pipeline enables SGCD to accurately distinguish interlayer boundaries in the DLPFC, clearly reflecting the layered structure and spatial evolution patterns of the cerebral cortex. Overall, SGCD demonstrates significant advantages in clustering accuracy and representation learning on the DLPFC dataset, while also excelling in hierarchical inference and spatial structure analysis, fully proving its practicality and superiority in tissue spatial data analysis.

### 2.3 SGCD is Capable of Precisely Characterizing and Analyzing the Structural Features of Complex Spatial Domains

**Figure 3:**
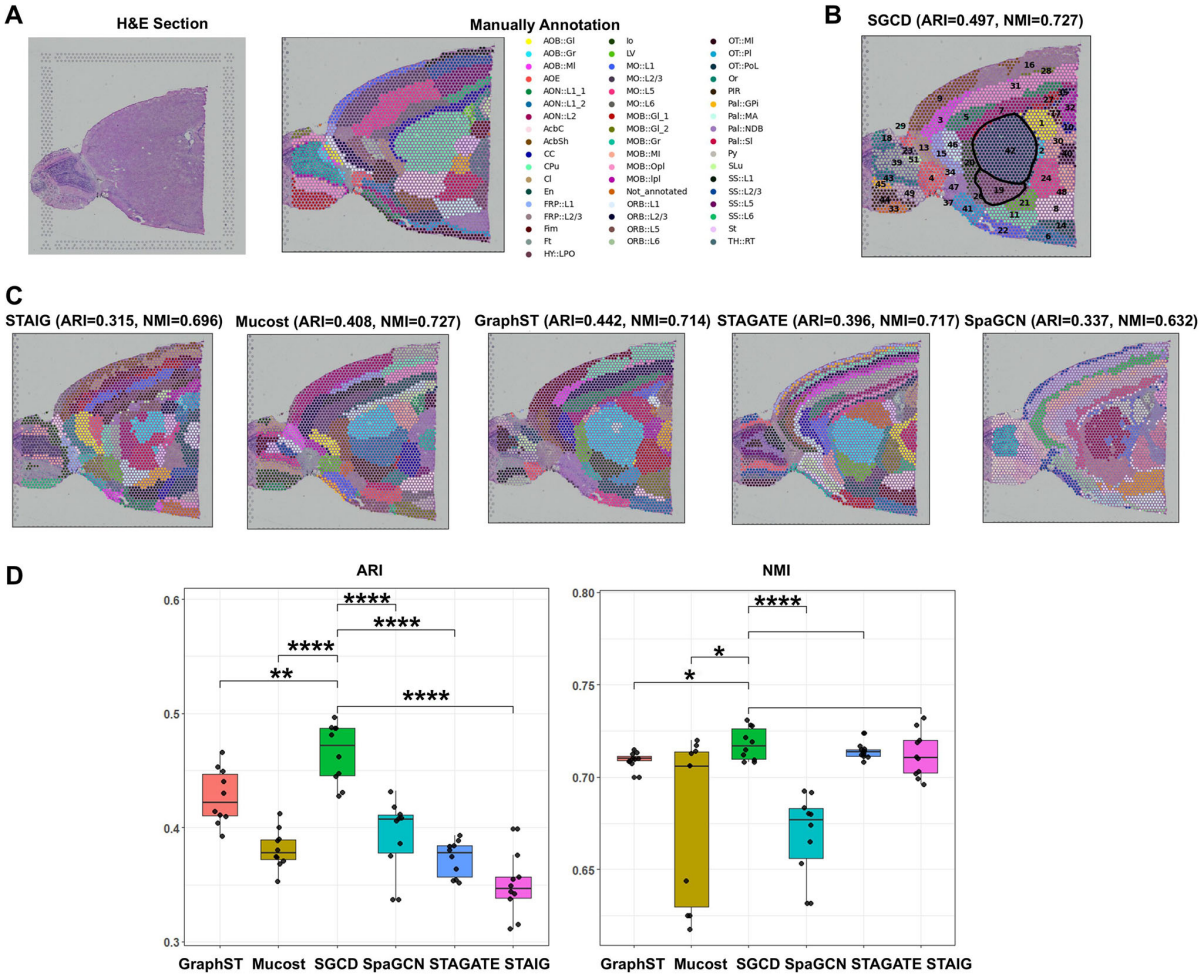
Application of SGCD on the mouse brain dataset. A) Histological image and manual annotation of mouse brain tissue. B) Clustering results of SGCD on the mouse brain dataset. C) Clustering results of STAIG, Mucost, GraphST, STAGATE, and SpaGCN. D) T‐test results to assess the significance of clustering results between SGCD and other methods.

To further validate the exceptional performance of SGCD in precisely characterizing and analyzing complex spatial domain structure features, we conducted an in‐depth evaluation of the structurally complex mouse brain slice dataset, which further demonstrates its significant advantages in complex spatial domain segmentation. This dataset contains 3,798 spots and 36,601 genes, divided into 52 subregions (**Figure** **3**A). We compared SGCD with SpaGCN, STAGATE, GraphST, MuCoST, and STAIG (Figure 3B,C). The results show that SGCD outperforms the other methods in clustering performance, with an ARI of 0.497 and an NMI of 0.727, surpassing the next best method, GraphST, by 11% in ARI score. Statistical results from t‐tests show that while SGCD's NMI score was not significantly better than those of STAGATE and STAIG, its ARI score was significantly superior to all other methods, with differences reaching significance levels of p < 0.01 (^**^) and p < 0.0001 (^****^) (Figure 3D).

Moreover, SGCD demonstrates exceptional ability in analyzing the complex spatial domains of the mouse brain. In the mouse brain, the CPu (striatum) is primarily involved in motor control, reward mechanisms, and habit formation, while the AcbC (nucleus accumbens core) is closely related to the regulation of executive functions, decision‐making, and reward mechanisms. SGCD identified spatial domains 42 and 19 as corresponding to CPu and AcbC, respectively (Figure 3B), with these divisions closely matching the true anatomical structure. In contrast, although STAGATE can capture the general contours, its detail rendering is not as precise as SGCD, while other methods show multiple spatial domain overlaps, making accurate and fine segmentation difficult.

The superior performance of SGCD stems from its deep integration of cell type information and spatial coordinates, constructing a weighted adjacency graph that simultaneously considers spatial distance and cell type similarity, endowing nodes with rich biological semantics. To address the gap issue in the regular hexagonal arrangement of 10X Visium data, SGCD employs gene expression data interpolation techniques to compensate for deficiencies in spatial continuity, finely characterizing spatial neighborhood relationships. This multidimensional information synergy significantly enhances the graph neural network's ability to capture local and global structural features, demonstrating unique advantages in complex spatial domain delineation and providing robust support for the precise analysis of brain microstructure and its functional regulatory mechanisms.

### 2.4 SGCD Enables Precise Analysis and Annotation of Spatial Domain Data Without Manual Labeling

**Figure 4:**
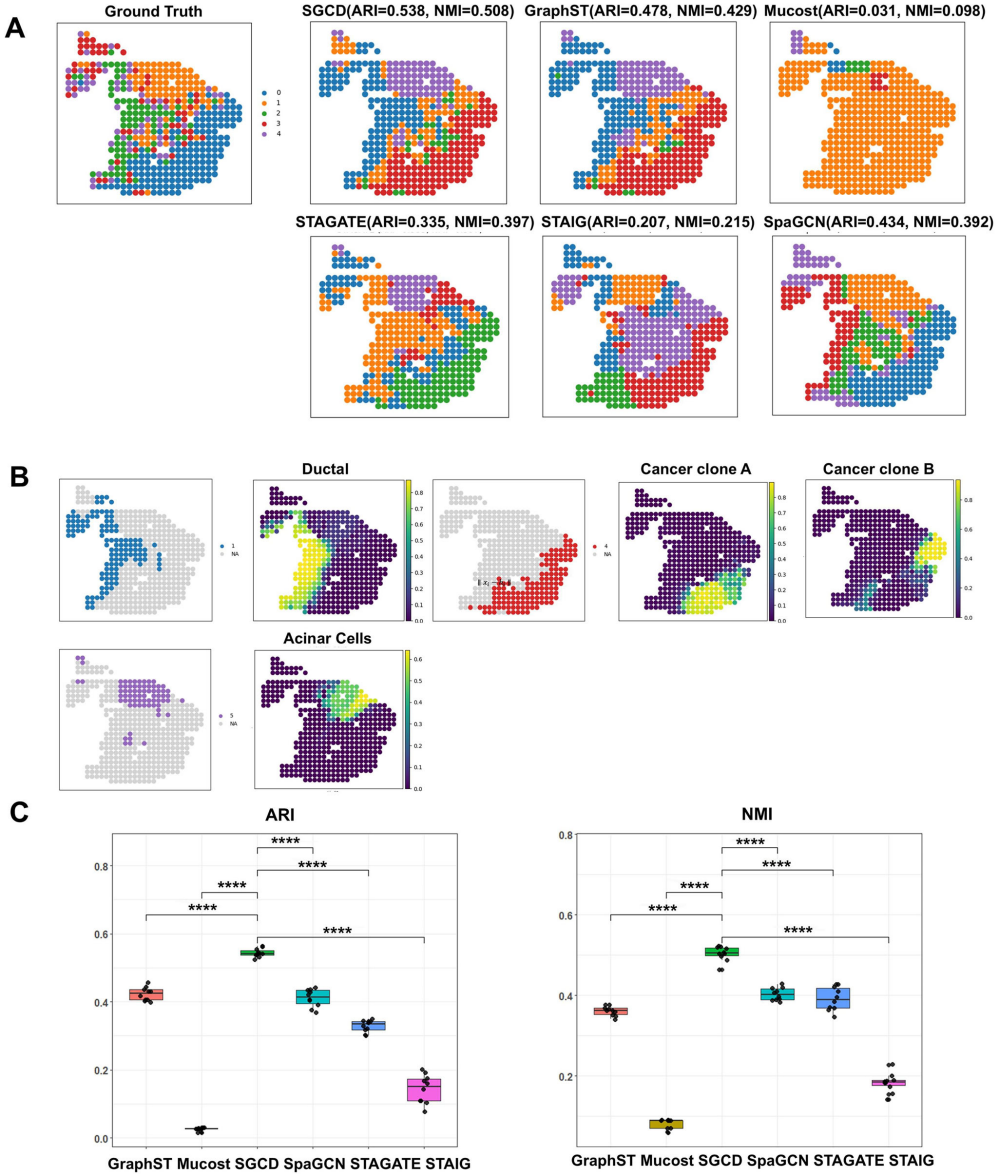
Application of SGCD on the PDAC‐A dataset. A) Clustering results of SGCD compared with STAIG, Mucost, GraphST, STAGATE, SpaGCN, and other methods on the PDAC‐A dataset. B) Spatial distribution of cell types in the PDAC‐A dataset using integrated single‐cell label data. C) T‐test results to assess the significance of clustering results between SGCD and other methods.

Pancreatic ductal adenocarcinoma (PDAC) is one of the most lethal malignancies worldwide, characterized by a highly complex and dynamic tumor microenvironment.^[^ ^32^ ^]^ To evaluate spatial clustering performance in this challenging context, we obtained benchmark data from the SDMBench database, where PDAC‐A and PDAC‐B(Figure S4, Supporting Information) tissue slices were each divided into five distinct regions. Here, we present the analysis results of the PDAC‐A slice (**Figure** **4**). Multiple spatial clustering methods were employed, including SpaGCN, STAIG, STAGATE, GraphST, MuCoST, and SGCD (Figure 4A). It is important to note that, due to compatibility issues with the PDAC dataset, SpaGCN and STAIG were analyzed without incorporating histological images.

Among all methods, SGCD exhibited the best overall performance, producing clear and highly accurate clustering results. SGCD achieved an Adjusted Rand Index (ARI) of 0.538 and a Normalized Mutual Information (NMI) score of 0.508, demonstrating strong clustering precision, particularly in distinguishing complex tissue regions. GraphST and SpaGCN also performed reasonably well, with ARI scores of 0.478 and 0.434, respectively, and were effective in delineating broader spatial domains. In contrast, STAGATE achieved an ARI of 0.335 and showed limitations in finer structural delineation, with evident region mismerging. While STAIG showed some advantages in global clustering, it lacked accuracy in fine‐grained segmentation. MuCoST performed the worst among all methods, with an ARI of just 0.031 and NMI of 0.098, failing to capture spatial structures in the data and proving unsuitable for the PDAC dataset. According to t‐test statistics, SGCD significantly outperformed all other methods, with differences reaching the highly significant level of p< 0.0001 (^****^) (Figure 4C).

Furthermore, to enrich spatial resolution, we employed a deconvolution strategy (using SpatialPrompt^[^ ^29^ ^]^ by default) to integrate single‐cell RNA‐seq data with interpolated spatial transcriptomics, enabling more detailed cell‐type composition for each spot (Figure 4B). Based on the spatial distribution of cell types, we conducted an in‐depth analysis of each spatial domain. In domain 0, the high‐density presence of cancer cell clones A and B indicated a typical cancerous region, reflecting tumor proliferation and malignancy. Domains 2, 3, and 4 were enriched with ductal epithelial cells but showed no signs of cancer cell proliferation, consistent with non‐malignant ductal epithelium. Domain 1, characterized by a high abundance of acinar cells and absence of cancer cells, corresponded to normal pancreatic tissue. These findings demonstrate SGCD's precision in distinguishing between malignant, non‐malignant epithelial, and normal tissue regions.

Of particular note is SGCD's suitability for datasets lacking ground‐truth annotations. Its high clustering accuracy enables effective inference of biological labels, offering a reliable spatial analysis tool for studying PDAC's tumor microenvironment. By leveraging SGCD, we not only achieved more precise spatial domain segmentation but were also able to infer biologically meaningful regional characteristics based on spatial and cellular context, even in the absence of annotated labels—providing a powerful foundation for disease diagnosis and therapeutic strategy development.

Building on the clustering results, we further investigated the factors contributing to SGCD's significant advantage. Most notably, SGCD constructs a weighted adjacency graph that integrates both cell‐type and spatial location information, effectively compensating for the spatial continuity gaps inherent in 10X Visium's sampling pattern. This multidimensional integration not only leads to superior clustering performance, as evidenced by ARI and NMI, but more importantly, enables automatic and accurate spatial domain inference in the absence of ground‐truth annotations. Consequently, SGCD shows unparalleled accuracy in distinguishing cancer regions, non‐malignant epithelial zones, and normal pancreatic tissue within PDAC, providing robust data support and theoretical insight for unraveling the functional mechanisms of the tumor microenvironment.

### 2.5 SGCD Unveils the Complex Spatial Patterns and Gene Expression Heterogeneity Within Cancer Tissues

**Figure 5:**
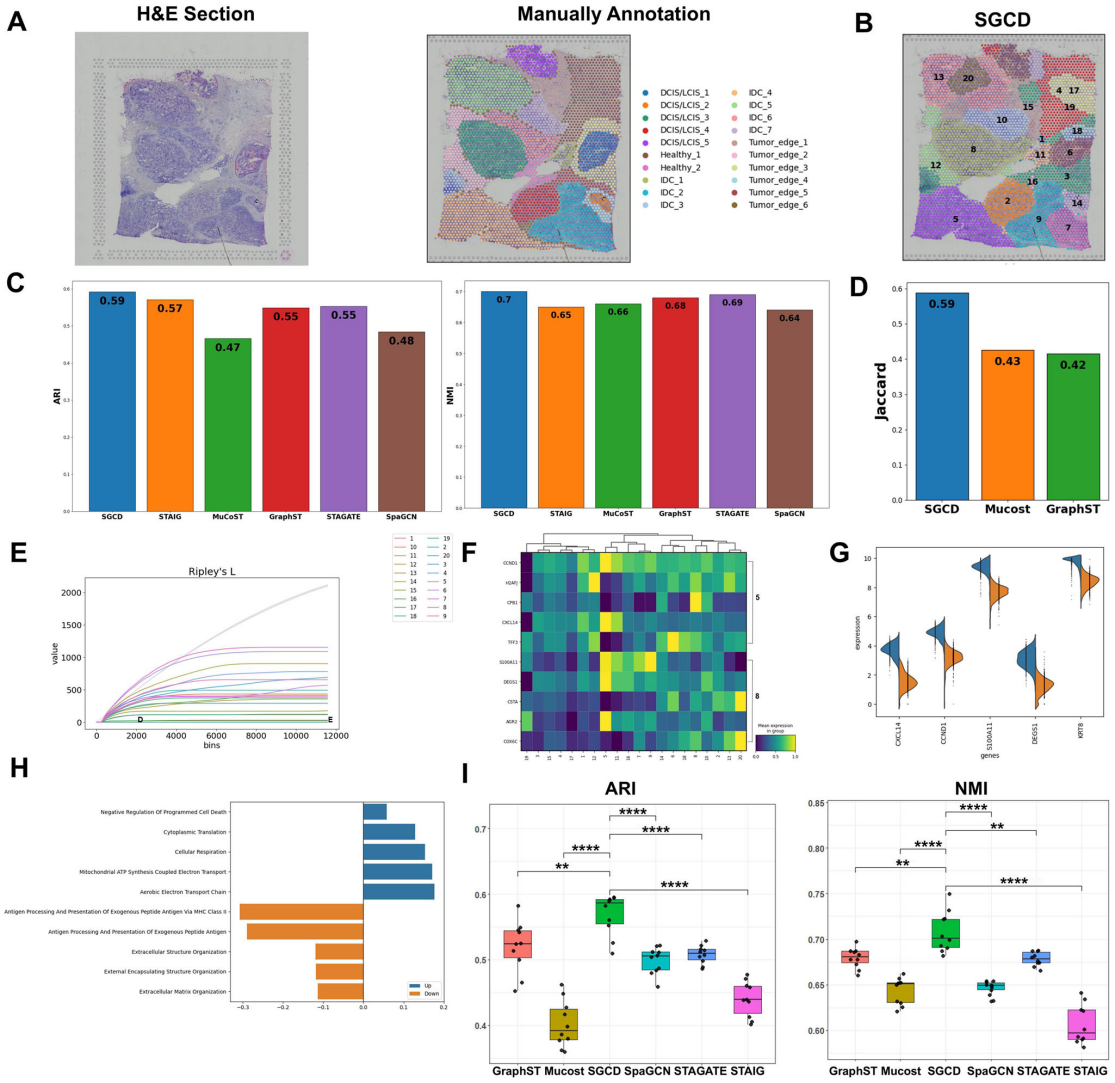
SGCD enhances the interpretability of complex and heterogeneous cancer tissues with functional spatial domains. A) Histological image and manual annotation results of breast cancer tissue slices. B) Spatial domain annotations by SGCD. C) Comparison of spatial domain recognition accuracy (ARI, NMI) between SGCD and five other methods. D) Jaccard index of spatial domains identified in the IDC_5 region by three methods compared to true annotations. E) Calculation of Ripley's L function for the point distribution of each spatial domain. F) Heatmap of gene expression profiles for the top five differentially expressed genes (DEGs) between spatial domain 5 and spatial domain 8 in the breast cancer dataset. G) Violin plot of the expression patterns of the top five differentially expressed genes between spatial domain 5 and spatial domain 8 in the breast cancer dataset. H) GO enrichment analysis between spatial domain 5 and spatial domain 8 in the breast cancer dataset. I) T‐test results to assess the significance of clustering results between SGCD and other methods.

Breast cancer is characterized by high heterogeneity and a complex microenvironment, making it difficult to fully describe its spatial structural features through manual annotations of tumor morphology. To address this, Fu et al. categorized histological images into four major morphological types and twenty subregions based on pathological features, including ductal carcinoma in situ/lobular carcinoma in situ (DCIS/LCIS), healthy tissue (Healthy), invasive ductal carcinoma (IDC), and tumor margins with low malignancy (**Figure** **5**A). To validate the generalization capability of SGCD in cancer tissues with complex spatial patterns and heterogeneous gene expression, we tested it on human breast cancer tissue data from 10X Visium (Figure 5B) and compared it with baseline methods, including STAIG, MuCoST, GraphST, STAGATE, and SpaGCN (Figure 5C; Figure S2, Supporting Information). The results showed that SGCD achieved the highest accuracy in spatial domain identification tasks (ARI = 0.59, NMI = 0.7). Statistical results from t‐tests revealed that SGCD significantly outperformed all other methods, with differences reaching significance levels of p < 0.01 (^**^) and p < 0.0001 (^****^) (Figure 5I).

To reveal the complex spatial patterns and highly heterogeneous gene expression features in breast cancer, we analyzed the spatial clustering results from two perspectives: spatial distance variation and spatially variable genes. First, using Ripley's L function, we computed the point distribution patterns for each spatial domain (Figure 5E). We found that compared to other more dispersed regions, spatial domains 8 (DCIS/LCIS_3), 5 (IDC_4), and 13 (IDC_5) exhibited significant clustering features. In the partitioning of DCIS/LCIS_3 and IDC_4, SGCD, STAIG, and GraphST consistently identified single spatial domains in line with reference results, while other methods showed multiple overlapping domains. For the partitioning of IDC_5, although no method fully aligned with the reference, SGCD clearly outlined the general contour of the region and only mixed with one other domain, while MuCoST and GraphST identified partial contours and mixed with more than two domains. The further computed Jaccard index (Figure 5D) showed that SGCD had the highest recognition accuracy.

To further investigate the heterogeneity of gene expression, we compared the top five differentially expressed genes between spatial domain 5 (representing the IDC region) and spatial domain 8 (representing the DCIS/LCIS region) (Figure 5F). The results revealed significant expression differences between the clusters, reflecting pronounced heterogeneity among tumor tissues. To gain deeper insights into the heterogeneous characteristics within tumor tissues, we conducted differential expression analysis and Gene Ontology (GO) enrichment analysis. Between spatial domains 5 and 8, a total of 359 significantly differentially expressed genes were identified (|log fold change| ≥ 2; *p*‐value < 0.05). Among the top five differentially expressed genes (Figure 5G), significant differences were observed in the expression levels of CXCL4, CCND1, S100A11, DEGS1, and KRT18. Notably, high expression of CXCL4 has been confirmed as an independent marker of poor prognosis in breast cancer,^[^ ^33^ ^]^ CCND1 plays a critical role in cell proliferation and is closely associated with tumor invasion and metastasis;^[^ ^34^ ^]^ S100A11 may promote tumor growth and metastasis by regulating signaling pathways in the tumor microenvironment;^[^ ^35^ ^]^ DEGS1 is involved in sphingolipid synthesis, and its metabolic dysregulation may accelerate tumor growth and spread by disrupting the balance between apoptosis and cell survival;^[^ ^36^ ^]^ high expression of KRT18 is associated with deep tumor infiltration, invasiveness, and drug resistance, and holds potential value in the diagnosis and prognostic evaluation of breast cancer.^[^ ^37^ ^]^ Interestingly, the high expression of CCND1 is closely linked to cell proliferation pathways, similar to the role of EGFR in clear cell renal cell carcinoma, where Lu et al. found that high EGFR expression drives tumor proliferation and is associated with poor prognosis.^[^ ^38^ ^]^ These findings suggest that breast cancer and renal cell carcinoma may share certain key proliferation signaling pathways, providing insights for cross‐cancer molecular mechanism studies.

Moreover, GO enrichment analysis of the differentially expressed genes (Figure 5H) revealed the biological processes (BP) that these genes were mainly involved in. The upregulated BPs were concentrated in immune regulation, cell proliferation, and macrophage activation processes, reflecting the cellular response to the tumor microenvironment or stress. Genes in spatial domain 5 were more clearly associated with tumor progression, invasion, and metastasis, while downregulated BPs primarily involved immune responses, indicating weakened cellular responses to inflammatory stimuli in spatial domain 8. The immune system's functions in handling exogenous antigens, extracellular matrix remodeling, and inflammatory regulation were suppressed. The weakened immune function could lead to tumor immune evasion, further promoting tumor cell survival and spread. These results are consistent with the study by Xu et al.,^[^ ^39^ ^]^ which found that in specific pathological states, diminished immune responses might suppress chronic inflammation and interfere with effective responses to exogenous pathogens, accelerating disease progression or leading to immune system dysfunction.

SGCD's outstanding performance in complex tumor microenvironments stems from its unique modeling pipeline. It first addresses the sampling gaps in 10X Visium data through interpolation, enhancing spatial continuity. Subsequently, it employs deconvolution techniques to extract precise cell type information, separating mixed signals. Finally, it constructs a weighted adjacency graph by integrating spatial coordinates with deconvolution results, capturing spatial neighborhood relationships and feature similarities among cells to achieve fine‐grained analysis of complex tumor spatial structures. This pipeline enables SGCD to accurately delineate spatial domains in breast cancer tissues, clearly revealing highly heterogeneous biological characteristics, and providing robust data support for analyzing tumor biological mechanisms, immune evasion, and precision therapy. In summary, leveraging its innovative modeling strategy, SGCD achieves the highest accuracy in spatial domain identification in breast cancer, effectively uncovering complex spatial patterns and gene expression heterogeneity within tumors. This demonstrates its unique advantage in handling complex microenvironments and offers a novel perspective for deepening the understanding of tumor biology.

### 2.6 Data Interpolation and Deconvolution Robustness Analysis

The spatial transcriptomics deconvolution process inevitably introduces uncertainty, exacerbated by missing gap information in low‐resolution data. We designed experiments to evaluate the effectiveness of the SGCD method in enhancing resolution and addressing deconvolution errors, by comparing performance before and after interpolation and quantifying model robustness under different noise levels.

#### 2.6.1 Impact of Interpolation on Deconvolution Accuracy

**Figure 6:**
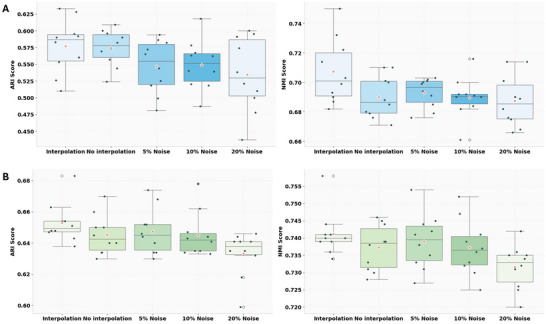
For data interpolation and deconvolution robustness analysis. A) Analysis of human breast cancer data. B) For slice analysis of DLPFC # 151673.

On both the breast cancer and DLPFC datasets (**Figure** **6**), the interpolation method improved SGCD's spatial domain identification performance. On the breast cancer dataset (Figure 6A), the median ARI of the interpolated method (0.59) was higher than that of the non‐interpolated method (0.57), and the NMI increased from 0.69 to 0.70. On the DLPFC dataset (Figure 6B), the median ARI of the interpolated method (0.65) was higher than that of the non‐interpolated method (0.64), with a more concentrated and stable performance distribution. This validates our hypothesis: interpolating gap regions enhances the continuity of spatial transcriptomics data, thereby improving the accuracy of cell‐type deconvolution.

#### 2.6.2 Noise Robustness of Deconvolution Results

We added uniformly distributed random noise of varying magnitudes (5%, 10%, 20%) to the deconvolution results to assess SGCD's robustness. On the breast cancer dataset (Figure 6A), as noise increased, the median ARI decreased from 0.59 to 0.55, 0.54, and 0.53. On the DLPFC dataset (Figure 6B), SGCD exhibited stronger robustness, with the median ARI decreasing only slightly from 0.65 to 0.64, 0.64, and 0.63, and the NMI decreasing from 0.74 to 0.73. Across both datasets, even at a high noise level of 20%, performance metrics remained at a high level, indicating SGCD's strong tolerance to noise.

In summary, SGCD enhances deconvolution accuracy through interpolation while exhibiting exceptional robustness to uncertainty in cell‐type deconvolution. Even under high‐noise conditions, SGCD maintains high‐precision spatial domain identification, providing a reliable tool for handling inevitable measurement errors in real biological samples.

### 2.7 Ablation Experiment

**Figure 7:**
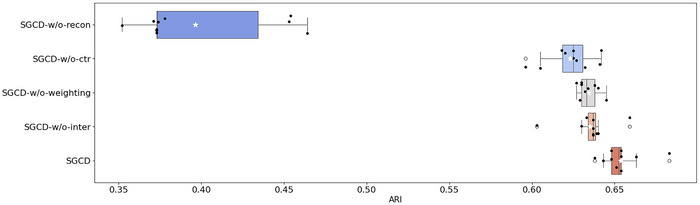
Boxplot of the SGCD ablation experiment results on the DLPFC dataset.

We conducted a series of ablation studies on the DLPFC dataset #151673 slice (**Figure** **7**) and the human breast cancer dataset (Figure S5, Supporting Information) to analyze and elucidate the functionality and mechanisms of SGCD.

- SGCD‐w/o‐recon refers to the model with the reconstruction loss module disabled.
- SGCD‐w/o‐ctr refers to the model with the contrastive loss module disabled.
- SGCD‐w/o‐weighting refers to the model with the weighted adjacency matrix disabled.
- SGCD‐w/o‐inter refers to the model with the data interpolation module disabled.

The ablation study results on the DLPFC dataset (Figure 7) demonstrate that the complete SGCD model significantly outperforms all ablation variants in terms of median ARI, confirming the positive contribution of each key module to overall performance. Specifically, disabling the reconstruction loss (SGCD‐w/o‐recon) led to a median ARI decrease of ≈42.8% compared to the full model, highlighting the critical role of reconstruction loss in capturing the internal structure of the data. In contrast, disabling only the contrastive loss module (SGCD‐w/o‐ctr) resulted in a median ARI reduction of ≈4.2%, reflecting the importance of contrastive loss in maintaining model stability and enhancing class discrimination. Additionally, disabling only the weighted adjacency matrix (SGCD‐w/o‐weighting) caused a median ARI drop of ≈2.4%, validating the advantage of incorporating cell type information through the weighted adjacency matrix to optimize spatial topology representation. Lastly, disabling the data interpolation module (SGCD‐w/o‐inter) similarly showed that the absence of interpolation negatively impacts the model's overall performance. Collectively, these experimental results further confirm the necessity and effectiveness of each component in the SGCD model.

The ablation study conducted on the breast cancer dataset (Figure S5, Supporting Information) further confirms the aforementioned findings. The consistent ablation results across the two distinct datasets robustly demonstrate the necessity and complementarity of each key component in the SGCD model, providing solid data support for the model's superior performance in complex spatial transcriptomics data analysis. These experimental results not only validate the rationality of the model design but also offer valuable references for future improvements in spatial transcriptomics data analysis methods.

### 2.8 Parameter Analysis

**Figure 8:**
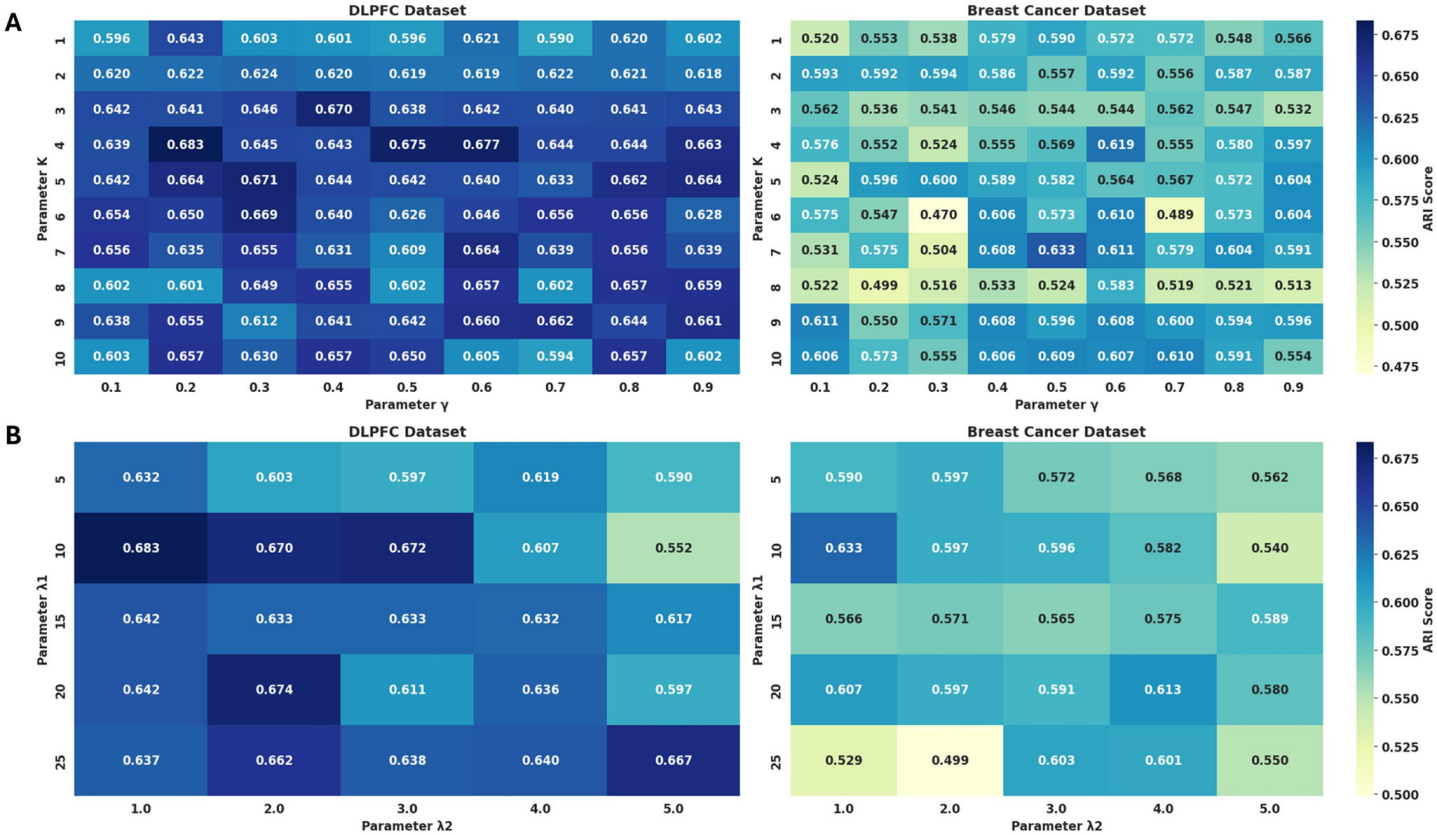
The performance of ARI indicators under different parameter combinations in two datasets. A) Analysis of neighbor number 𝐾 and spatial weight *γ* on DLPFC section # 151673 and breast cancer data set. B) Analysis of reconstruction loss weight ***λ***_1_ and comparison loss weight ***λ***_2_ on DLPFC and breast cancer data sets.

We further investigated the impact of four key parameters in the model—number of neighbors 𝐾, spatial weight *γ*, and reconstruction loss weight. λ_1_, and contrastive loss of weight λ_2_—on model performance. Given that other parameters adopt default settings from prior studies, this study focuses solely on the rational selection of these four parameters and their sensitivity analysis for SGCD model performance. As shown in **Figure** **8**A, with the number of neighbors 𝐾 on the vertical axis and spatial weight *γ* on the horizontal axis, the heatmap illustrates the ARI metric under different parameter combinations. In both datasets, we fixed the reconstruction loss weight λ_1_ = 10 and contrastive loss of weight λ_2_ = 1, analyzing the impact of 𝐾 and *γ* on clustering performance (ARI metric) through heatmap visualization. The results show that model performance is optimal when both 𝐾 and *γ* are in a moderate range, indicating the need for a balance between local spatial information and cell type information. Specifically, appropriately increasing 𝐾 helps capture more local spatial information, but an excessively large 𝐾 introduces distant neighbors, leading to over‐smoothing of local features and blurring clustering boundaries. Meanwhile, *γ* controls the trade‐off between spatial information and cell‐type information: a higher *γ* emphasizes spatial information while relatively weakening cell‐type information, whereas a lower *γ* enhances sensitivity to cell‐type information. Based on comprehensive consideration of both datasets, selecting 𝐾 between 3 and 7 and *γ* ≈0.2 to 0.5 significantly improves model performance in spatial transcriptomics data clustering tasks by leveraging spatial information while maintaining sensitivity to cell type information.

As shown in Figure 8B, with the reconstruction loss weight λ_1_on the vertical axis and contrastive loss of weight λ_2_ on the horizontal axis, the heatmap illustrates the ARI metric under different parameter combinations. For the DLPFC dataset, we fixed 𝐾 = 4 and *γ* = 0.2, and for the breast cancer dataset, we fixed 𝐾 = 7 and *γ* = 0.5. The heatmap shows that in both datasets, model performance is optimal when λ_1_ = 10 and λ_2_ = 1. Considering both stability and model generalization, we recommend setting λ_1_ = 10 and λ_2_ = 1, which ensures synergistic optimization of reconstruction and contrastive tasks while achieving the best clustering performance.

### 2.9 Computational Complexity and Hardware Requirements Analysis

To evaluate the computational performance of our method, we conducted tests on processing time and memory consumption across datasets of varying scales. Figure S7 (Supporting Information) presents the test results on an NVIDIA A100 80GB GPU. As shown in Figure S7A (Supporting Information), the processing time is closely related to the number of spatial points in the dataset. The largest DLPFC dataset (4,788 spatial points) requires ≈27 min, while the smaller PDAC dataset takes only tens of seconds. Figure S7B (Supporting Information) indicates that even the largest dataset consumes only ≈3GB of memory, accounting for less than 4% of the A100 GPU's total memory (80GB). This demonstrates that our method is highly efficient in memory usage, making it suitable for processing large‐scale datasets. Through testing, we found that the algorithm can smoothly handle medium‐scale datasets (≈5,000 spatial points) on modern GPUs with 8GB or more of memory. For larger‐scale datasets, our method achieves efficient processing through spatial graph construction and spatial interpolation techniques. It is estimated that, with optimization, it can handle large‐scale datasets exceeding 50,000 spatial points while maintaining reasonable computational resource demands.

## 3 Conclusion

In this study, we address the challenges of missing information and insufficient cellular composition in spatial domain recognition tasks within low‐resolution spatial transcriptomics data. We propose the SGCD method, which achieves significant advancements in spatial domain recognition through graph contrastive learning to facilitate multi‐dimensional information fusion. Traditional spatial domain recognition methods typically rely on the proximity assumption between points, focusing primarily on overall expression patterns while neglecting potential gene expression and cell type information in gap regions. To overcome this, SGCD first utilizes interpolation techniques to preprocess the original 10X Visium data, effectively filling the gaps between sampling points and improving spatial continuity. Subsequently, a deconvolution method is applied to extract cellular composition information, ensuring that each spot contains not only gene expression data but also rich biological context.

Based on this, we construct a weighted adjacency matrix that integrates both spatial position and cell type information, with the weight between the two being dynamically adjusted by a parameter, γ, to finely characterize local spatial relationships. Finally, through the graph contrastive learning framework, we distill low‐dimensional representations, ensuring that spatially adjacent regions with similar cell types are embedded more consistently. This multi‐layered, multi‐dimensional information fusion strategy not only breaks through the limitations of traditional methods that rely on a single source of information but also provides new theoretical and practical insights into the application of graph neural networks in spatial transcriptomics.

The validation across multiple public datasets demonstrates SGCD's superior performance. On the human dorsolateral prefrontal cortex (DLPFC) dataset, SGCD outperformed existing methods in clustering accuracy across all 12 slices, achieving an average ARI of 0.651 and NMI of 0.710. On the structurally complex mouse brain dataset, faced with the task of delineating 52 fine‐grained spatial domains, SGCD achieved an ARI of 0.497 and NMI of 0.727, accurately identifying known regions while also capturing finer subregional structures. On the pancreatic ductal adenocarcinoma (PDAC) dataset, SGCD precisely resolved complex spatial domain structures in the absence of ground‐truth labels. In its application to the breast cancer dataset, SGCD successfully captured the biological characteristics of different tumor regions through detailed spatial domain delineation. By integrating spatial distance, spatially variable genes, and differential expression analysis, SGCD deeply revealed significant differences in key biological processes such as immune regulation and cell proliferation across regions, providing a powerful tool for studying tumor heterogeneity.

SGCD's core innovation lies in its unique solution to the gap issue in low‐resolution data. First, through interpolation techniques, it effectively compensates for information loss between sampling points, restoring data continuity and offering a transferable approach for addressing similar gap issues in other low‐resolution datasets. Second, the weighted adjacency matrix, constructed by integrating cell type and spatial location information, overcomes the limitations of traditional methods that rely solely on spatial proximity, enabling more accurate spatial domain delineation. This approach demonstrates significant potential in resolving complex biological structures, providing new insights for tumor microenvironment analysis, brain region stratification, and related fields.

Although the current SGCD framework is primarily designed for single‐slice analysis, it exhibits excellent scalability. For multi‐slice extension, we propose using registration algorithms to ensure spatial alignment of slices, constructing a joint neighborhood graph that incorporates both intra‐ and inter‐slice relationships; applying batch effect correction strategies for slices from different batches; designing a hierarchical contrastive learning framework to capture spatial relationships within and across slices; and introducing a slice identifier mechanism to enable complete reconstruction of 3D spatial structures, thereby extending SGCD from 2D planar analysis to 3D volumetric analysis.

With the advancement of spatial omics technologies, SGCD can be extended into a multimodal version, integrating spatial location, cell type, and inter‐modal correlations, with weights dynamically adjusted through learnable parameters and cross‐modal contrastive learning. These extensions will significantly enhance SGCD's application scope and biological insight capabilities, offering a more comprehensive computational analysis framework for the systematic characterization of tissue microenvironments and disease mechanism studies.

## 4 Experimental Section

### Data Preprocessing

For scRNA‐seq data preprocessing, the raw gene expression counts were first log‐transformed and normalized by library size, followed by scaling to unit variance and zero mean. To effectively retain biologically significant differential information while reducing the computational complexity associated with high‐dimensional data, this study selected the top 3000 highly variable genes (HVGs) for subsequent analysis, based on current mainstream preprocessing methods.^[^ ^40^ ^]^ For ST‐seq data, in order to maintain consistency with the scRNA‐seq data processing pipeline, a similar preprocessing approach was applied. The raw gene expression counts were log‐transformed and normalized by library size. The standardized gene expression counts were then scaled to unit variance and zero mean. Finally, to highlight biological differences in spatial expression and reduce computational complexity, the ST‐seq data also selected the top 3000 highly variable genes for use as input into the model.

### Data Interpolation and Completion

The method presented in this paper was based on data from the 10X Visium platform, where the sampling points were arranged in a regular honeycomb pattern. Therefore, the entire tissue section was divided into rows along the horizontal direction, and in each row, segmentation was performed along the left and right 45° diagonals, thereby creating a complete gap region between the three points. Subsequently, based on the original spatial coordinate data, the centroid of the divided regions was used as the new spatial coordinates for the gap regions. The method for calculating the centroid coordinates is as follows Equations (1) and (2):

(1) $$x_{centroid}=\frac{1}{N}\sum_{i=1}^{N}x_{i}$$

(2) $$y_{centroid}=\frac{1}{N}\sum_{i=1}^{N}y_{i}$$

here, (*x_i_*,*y_i_*)represents the coordinates of the points within each segmented region, and *Ｎ* is the total number of points within the region.

Gene expression data interpolation was performed for the segmented gap regions based on the potential relationship between gene expression and spatial coordinates in the original spatial transcriptomic data. The neighborhood‐based interpolation method is expressed as follows in Equation (3):

(3) $$g\left(x,y\right)=\sum_{i=1}^{N}\alpha_{i}g_{i}$$

here, the gene expression at the spatial coordinate (*x*, *y*) is denoted as *g*(*x*, *y*), α_*i*_ is the coefficient calculated based on spatial distance and other weighting factors, and *g_i_* is the gene expression value of other points within the neighborhood. Here, the gene expression at spatial coordinates (*x*, *y*) is denoted as *g*(*x*, *y*), and the weight coefficient α_*i*_ is calculated based on the Cauchy kernel function in the SpaVAE model:

(4) $$\alpha_{i}=\frac{k_{\theta }\left(x_{new},x_{i}\right)}{\sum_{j=1}^{N}k_{\theta }\left(x_{new},x_{j}\right)}$$

The Cauchy kernel function is defined as:

(5) $$k_{\theta }\left(x_{i},x_{j}\right)=\frac{1}{1+\frac{{\left\|x_{i}-x_{j}\right\|}^{2}}{\theta }}$$

The parameter *θ* is a trainable spatial dependency strength parameter, ‖*x_i_* − *x_j_* ‖^2^ represents the Euclidean distance between spatial locations. *g_i_* denotes the gene expression values of other points within the neighborhood. This interpolation method based on Gaussian processes ensures spatial continuity and smoothness.

The mathematical derivation of the interpolation method was based on the Gaussian process framework of SpaVAE. Specifically, the relationship between spatial coordinates and gene expression is established as follows:

Latent Space Modeling: The gene expression 𝑦 and spatial coordinates 𝑥 are associated through a latent variable 𝑧, where the first *L* dimensions of the latent variable follow a Gaussian process prior:

(6) $$p\left(z_{1:L}|x\right)=GP\left(0,K_{NN}\right)$$

The covariance matrix *K_NN_* is computed based on the spatial coordinates 𝑥, with its elements defined as:

(7) $${\left[K_{NN}\right]}_{ij}=k_{\theta }\left(x_{i},x_{j}\right)$$

The latent representation for a new location *x_new_* is obtained through Gaussian process regression as follows:

(8) $$p\left(z_{new}|z_{1:N},x_{1:N},x_{new}\right)=N\left(\mu_{new},{\sigma^{2}}_{new}\right)$$

wherein:

(9) $$\mu_{new}=k_{*}^{T}K_{NN}^{1}z_{1:N}$$

(10) $${\sigma^{2}}_{new}=k_{\theta }\left(x_{new},x_{new}\right)-k_{*}^{T}K_{NN}^{-1}k_{*}$$

(11) $$k_{*}={\left[k_{\theta }\left(x_{new},x_{1}\right),\text{…},k_{\theta }\left(x_{new},x_{N}\right)\right]}^{T}$$

Gene expression reconstruction:

(12) $$g\left(x_{new}\right)=decoder\left(z_{new}\right)$$

The trained decoder maps the latent representation back to the gene expression space.

### Construction of Spatial Adjacency Graph

A unique advantage of spatial transcriptomics lies in its ability to capture not only gene expression information but also spatial location data. This enables the identification of regions that were spatially adjacent and share similar cellular states, thus facilitating the delineation of subdomains within the tissue. To fully leverage this spatial information, the data was converted into an undirected graph, where node *Ｖ* represents all the sampling points (spots), and edge *Ｅ* represents the connections between these points.

First, the Euclidean distance between each point and every other point was calculated based on their spatial coordinates, resulting in a distance matrix that reflects the physical proximity between points. Next, using the k‐nearest neighbors (KNN) strategy (with 𝐾 = 4 in the experiment), the 𝐾 nearest points to each point were selected as its neighbors. This creates an initial adjacency matrix, where a value of 1 indicates that point 𝑗 is a neighbor of point 𝑖, and 0 otherwise. To ensure the symmetry of the graph, the original adjacency matrix is added to its transpose and values greater than 1 are truncated to 1, making all connections binary (either 0 or 1).

To further enhance the expressive power of the graph model, cell‐type information was incorporated. It was assumed that points that were spatially adjacent and have similar cell types were more likely to belong to the same spatial domain, and therefore assign higher weights to existing edges. Specifically, for any connected spot pair (*i*, *j*), their cell type similarity *S_ij_* was calculated.

This study provides three methods for calculating cell type similarity: Jensen–Shannon Divergence (JSD), cosine similarity, and Pearson correlation coefficient. Experimental comparisons (Figure S6, Supporting Information) show that the performance differences among these three methods were minor, but JSD slightly outperformed the others, making it the default choice. The specific formulas are as follows:

(13) $$S_{ij}=\frac{1}{1+Sim}$$

The similarity *Sim* represents the similarity calculated using the three methods.

The Jensen–Shannon Divergence (JSD) is a symmetric form of the Kullback–Leibler (KL) divergence. Let *P* and *Q* represent the probability distributions of the predicted and true results, respectively. The JSD can be expressed as:

(14) $$JSD\left(P∥Q\right)=\frac{1}{2}D_{KL}\left(P∥\frac{1}{2}\left(P+Q\right)\right)+\frac{1}{2}D_{KL}\left(Q∥\frac{1}{2}\left(P+Q\right)\right)$$

The Kullback‐Leibler (KL) divergence *D_KL_* can be expressed as:

(15) $$D_{KL}\left(P∥Q\right)=\sum_{x\varepsilon X}P\left(x\right)\log\frac{P\left(x\right)}{Q\left(x\right)}$$

The cosine similarity can be expressed as:

(16) $$cosine\left(A,B\right)=\frac{A\cdot B}{\left|A\right|\left|B\right|}$$

The probability vectors *A* and *B* represent the probability distributions of two points, respectively.

The Pearson correlation coefficient can be expressed as:

(17) $$S_{ij}=\frac{\sum_{k=1}^{m}\left(P_{i}\left(k\right)-{\bar{P}}_{i}\right)\left(P_{j}\left(k\right)-{\bar{P}}_{j}\right)}{\sqrt{\sum_{k=1}^{m}{\left(P_{i}\left(k\right)-{\bar{P}}_{i}\right)}^{2}}\cdot \sqrt{\sum_{k=1}^{m}{\left(P_{j}\left(k\right)-{\bar{P}}_{j}\right)}^{2}}}$$

The term *m* represents the total number of cell types, and *P_i_* (*k*) denotes the proportion of the i‐th sample in the k‐th cell type.

Finally, the edge weights are updated according to Equation (18).

(18) $$weighted_{value}=\gamma +\left(1-\gamma \right)\times S_{ij}$$

here, *γ* serves as the baseline weight for spatial information, ensuring that the edge weight did not fall below *γ*, even when the cell type similarity is low. *γ* was defaulted to 0.2. This dual constraint‐based weighting mechanism, incorporating both spatial distance and cell type similarity, allowed the graph neural network to better integrate these two types of information during feature extraction. As a result, spatially adjacent points with similar cell types become more closely associated in feature space, enabling more accurate spatial domain segmentation.

### Gene Expression Random Graph Construction

Given the original neighborhood graph *G*  =  (*V*, *E*) and the normalized gene expression matrix *Ｘ*, a technique similar to the one used in the GraphST^[^ ^25^ ^]^ method was adopted, applying a form of perturbation to generate a random graph *G*′  =  (*V*′, *E*′) and its corresponding expression matrix *Ｘ’*, while keeping the graph's topological structure unchanged. Specifically, the original coordinates of spatial spots were maintained unchanged, while only the gene expression data was perturbed. The perturbation of gene expression vectors was implemented through random permutation. This perturbation method randomly rearranged the order of spots in the entire gene expression matrix, essentially shuffling the correspondence between spots and gene expression vectors. During model training, random permutations in each epoch were regenerated.

### GCN‐Based Encoder

In SGCD, the fundamental principles of Graph Convolutional Networks (GCN) were adopted by modeling the data as a graph *G*  =  (*V*, *E*), where the node set *Ｖ* includes all sampling points (spots), and the edge set *Ｅ* represents the connections between nodes. Specifically, let $X\varepsilon R^{N_{spot}\times d}$ denote the node feature matrix, where *N_spot_* is the number of nodes and d is the dimensionality of each node's features. The adjacency matrix $A\varepsilon R^{N_{spot}\times N_{spot}}$ encodes the relationships between nodes, which can either be a weighted matrix or a binarized adjacency matrix, reflecting the connectivity among nodes.

To perform graph convolution, the adjacency matrix *A* was first normalized to obtain the normalized adjacency matrix $\tilde{A}=D^{-\frac{1}{2}}AD^{-\frac{1}{2}}$, where *D* is a diagonal matrix with diagonal elements $D_{ii}=\sum_{j=1}^{N_{spot}}A_{ij}$, representing the degree of node *i*. This normalization step helped mitigate the impact caused by the differences in node degrees during the graph convolution operation.

In the l‐th layer of the graph convolutional network, the update rule for node features could be expressed as Equation (19):

(19) $$Z_{s}^{l}=\sigma \left(\tilde{A}Z_{s}^{l-1}W_{e}^{l}+b_{e}^{l}\right)$$

where $Z_{s}^{l}\in R^{N_{spot}\times d_{l-1}}$ is the output of the (l‐1)‐th layer (i.e., the node feature representation from the previous layer), $W_{e}^{l}\in R^{d_{l-1}\times d_{l}}$ and $b_{e}^{l}\in R^{d_{l}}$ are the trainable weight matrix and bias term of the l‐th layer, respectively, and σ(·) is the nonlinear activation function (e.g., ReLU). Finally, $Z_{s}^{l}$ represents the output of the l‐th layer, and we set $Z_{s}^{0}$ to be the original input gene expression matrix *Ｘ*. *Z_s_* as the final output of the encoder was taken, where each row *Z_i_* ​ represents the latent representation of sampling point *i*.

Next, the latent representations *Z_s_* were fed into the decoder, which then mapped them back to the original gene expression space through the decoding process. Unlike the encoder, the decoder employs a symmetric structure to reconstruct the gene expression profile. The specific structure of the decoder is as follows in Equation (20):

(20) $$H_{s}^{t}=\sigma \left(\tilde{A}H_{s}^{t-1}W_{d}^{t-1}+b_{d}^{t-1}\right)$$

where $H_{s}^{t}$ denoted the reconstructed gene expression profile generated at the t‐th layer, and $H_{s}^{0}$ was initialized with the encoder's output *Z_s_*. *W_d_* and *b_d_* represented the trainable weight matrix and bias vector in the decoder, respectively, and were shared across all nodes.

To effectively train the model, its parameters were optimized by minimizing the self‐reconstruction loss of the gene expression profiles. Specifically, the reconstruction loss function is defined as follows in Equation (21):

(21) $$L_{recon}=\sum_{i=1}^{N_{spot}}∥x_{i}-h_{i}{∥}_{F}^{2}$$

here, *x_i_* and *h_i_* represent the original normalized gene expression profile and the reconstructed gene expression profile of node *i*, respectively. By minimizing this loss function, the model learns effective node representations that allowed accurate reconstruction of gene expression profiles from the latent space.

In this way, SGCD effectively models gene expression data while leveraging the structural information captured by the graph convolutional network, thereby enhancing performance in the gene expression reconstruction task.

### Self‐Supervised Contrastive Learning Module

To enhance the representational power of graph embeddings and improve the discriminative ability between nodes, a strategy based on Self‐Supervised Contrastive Learning (SCL), aimed at extracting richer feature information from the local spatial context was proposed. The core idea of this strategy was to optimize the representation power of Graph Convolutional Networks (GCNs) through self‐supervised learning, enabling each node's representation to better capture its neighborhood structure and microenvironment features.

Specifically, the original graph *Ｇ* and a random graph *Ｇ’* was used as inputs, and employed the Graph Convolutional Network (GCN) encoder to generate two representation matrices *Z_s_* and *Z_s_* ′, respectively. The task of the GCN encoder was to aggregate the neighboring information of each node to generate the node embedding, thereby forming the local context vector *g_i_* for each node. This vector represented the semantic information of the node within its local environment, obtained by aggregating the representations of its neighboring nodes.

In this design, positive and negative sample pairs were constructed: the representation *Z_i_* of node *i* and its local context *g_i_* form a positive sample pair, while in the random graph *Ｇ’*, the representation *Z_i_* ′ of node *i* and its context *g_i_* form a negative sample pair. The positive sample pair aimed to maintain the similarity between the node and its local environment, while the negative sample pair helped the model differentiate the semantic differences between nodes.

The objective of SCL was to optimize the quality of node representations by maximizing the mutual information between positive sample pairs and minimizing the mutual information between negative sample pairs. In this way, the representations of neighboring nodes became more similar, while different nodes were effectively distinguished. To achieve this, a binary cross‐entropy (BCE) loss function was used to construct a contrastive loss function, which is formulated as follows in Equation (22):

(22) $$\begin{array}{ccc} L_{SCL} & = & -\frac{1}{2N_{spot}}\sum_{i=1}^{N_{spot}}\left(E_{\left(X,A\right)}\left[\log\Phi \left(z_{i},g_{i}\right)\right]\right.\\ & & \left.+\;E_{\left(X^{\prime },A^{\prime }\right)}\left[\log\left(1-\Phi \left({z^{\prime }}_{i},g_{i}\right)\right)\right]\right) \end{array}$$

where Φ(·) is a discriminator *D*, defined as $D:R^{d}\times R^{d}\to R$, consisting of a dual neural network designed to distinguish positive and negative sample pairs. Φ(*z_i_*,*g_i_*) represents the probability score of the positive sample pair (*z_i_*,*g_i_*).

Additionally, to improve model stability and reduce the influence of noise during training, a symmetric contrastive loss $L_{SCL\_random}$ for *Ｇ’* was introduced. By incorporating the construction of the random graph, the training process became more balanced in terms of positive and negative sample pairs, preventing overfitting to certain graph topologies. The form of this contrastive loss is as follows in Equation (23):

(23) $$\begin{array}{ccc} L_{SCL_{random}} & = & -\frac{1}{2N_{spot}}\sum_{i=1}^{N_{spot}}\left(E_{\left(X^{\prime },A^{\prime }\right)}\left[log\Phi \left({z^{\prime }}_{i},{g^{\prime }}_{i}\right)\right]\right.\\ & & \left.+\;E_{\left(X,A\right)}\left[\log\left(1-\Phi \left(z_{i},{g^{\prime }}_{i}\right)\right)\right]\right) \end{array}$$

In this way, SCL could dynamically adjust the model's learning objective during training, ensuring that the model not only learns the local relationships between nodes but also maintains a certain level of robustness across different graph structures. Ultimately, the contrastive learning‐based loss function helped the model progressively optimize node representations, allowing it to better capture the complex structural information in graph data.

Overall, this method leveraged the advantages of self‐supervised contrastive learning to not only improve the accuracy of node representations but also enhanced the model's stability and generalization ability when handling complex graph data. In various graph data tasks, especially when the structural relationships between nodes were complex and the semantic information of nodes was rich, the SCL method could effectively improve the learning performance of graph neural networks and provide strong support for further graph data analysis.

### Loss Function

For the loss function $L_{total}$, it was divided into two main components: the self‐reconstruction loss and the contrastive loss. The self‐reconstruction loss $L_{recon}$ represented the reconstruction error between the original gene expression data and the reconstructed data. The contrastive loss consisted of $L_{SCL}$ for positive sample pairs and $L_{SCL_{random}}$ for negative sample pairs. The representation learning module for spatial transcriptomic data was trained by minimizing both the self‐reconstruction loss and the contrastive loss. In summary, the overall training loss for this module is defined as Equation (24):

(24) $$L_{total}=\lambda_{1}L_{recon}+\lambda_{2}\left(L_{SCL}+L_{SCL_{random}}\right)$$

where λ_1_and λ_2_ are weighting factors that balance the influence of the reconstruction loss and the contrastive loss. Empirically, we set λ_1_ = 10 and λ_2_ = 1.

### Clustering

The trained latent representations were combined with R's mclust^[^ ^41^ ^]^ algorithm and Scanpy's^[^ ^40^ ^]^ Leiden and Louvain algorithms for preliminary partitioning to obtain different spatial domains. For the mclust algorithm, the ‘EEE’ model was selected, which performed stably in spatial transcriptomic data and did not make overly strong assumptions about cluster shapes. Before applying clustering, first reduce the latent representations to 20 dimensions through PCA, balancing information retention and noise reduction effects. For Leiden and Louvain clustering algorithms, an adaptive parameter selection strategy was adopted, not using fixed resolution values. A graph based on the 50 nearest neighbors was constructed, then automatically searched for the optimal resolution value that produces the target number of clusters within the resolution range of 0.1–3.0 with a step size of 0.01. This approach avoided the tedious process of manually adjusting resolution and ensured consistency of results across different datasets. Following this, category labels are reassigned using the refine function to ensure consistency with the majority of default neighbors in the spatial graph, leading to improved partitioning detail. The refining process considered the label distribution within a spatial neighborhood of radius 50 for each point, updating the point's label to the most common label in the neighborhood. The refining operation was performed for data with real labels while maintaining initial clustering for other data. This hyperparameter strategy performed consistently across all test datasets without requiring separate parameter adjustments for each dataset.

After clustering, PAGA in Scanpy was used to infer spatial trajectories. To decode the spatial architecture of tissue slices, Scanpy's spatial visualization tools were leveraged. Ripley's L function was employed from Squidpy to calculate spatial aggregation or differential patterns across the spatial domains.^[^ ^42^ ^]^ Additionally, Scanpy's gene ranking tool was used to identify genes that vary spatially. Furthermore, the upregulated signaling pathways were visualized in functional spatial domains through GO enrichment analysis using GSEApy.^[^ ^43^ ^]^

### Evaluation Metrics

The clustering results were evaluated using two commonly used clustering metrics: the Adjusted Rand Index (ARI)^[^ ^44^ ^]^ and Normalized Mutual Information (NMI).^[^ ^45^ ^]^ As shown in Equation (25):

(25) $$ARI=\frac{\sum_{ij}\left(\begin{array}{c} n_{ij}\\ 2 \end{array}\right)-\frac{\left[\sum_{i}\left(\begin{array}{c} a_{i}\\ 2 \end{array}\right)\sum_{j}\left(\begin{array}{c} b_{j}\\ 2 \end{array}\right)\right]}{\left(\begin{array}{c} n\\ 2 \end{array}\right)}}{\frac{1}{2}\left[\sum_{i}\left(\begin{array}{c} a_{i}\\ 2 \end{array}\right)+\sum_{j}\left(\begin{array}{c} b_{j}\\ 2 \end{array}\right)\right]-\frac{\left[\sum_{i}\left(\begin{array}{c} a_{i}\\ 2 \end{array}\right)\sum_{j}\left(\begin{array}{c} b_{j}\\ 2 \end{array}\right)\right]}{\left(\begin{array}{c} n\\ 2 \end{array}\right)}}$$

here, *a_i_* and *b_j_* represent the number of samples in the i‐th predicted cluster and the j‐th ground truth cluster, respectively; *n_ij_* denotes the number of overlapping samples between the i‐th predicted cluster and the j‐th ground truth cluster.

(26) $$NMI\left(Y,C\right)=\frac{2\;\times \left[H\left(Y\right)-H\left(Y|C\right)\right]}{\left[H\left(Y\right)+H\left(C\right)\right]}$$

here, *C* and *Y* denote the predicted clustering and the ground truth clustering, respectively, and the function *H*(·) is used to compute the entropy.

### Benchmarking Methodology

To assess the performance of the approach, SGCD was compared with several other tools, including STAIG, Mucost, GraphST, STAGATE, and SpaGCN. All competing methods were tested on datasets using the default hyperparameters and preprocessing recommended in the original papers. All the methods were evaluated based on the number of true clusters available for clustering in the datasets.

## Conflict of Interest

The authors declare no conflict of interest.

## Author Contributions

T.Z. and S.L. contributed equally to this work. G.W. conceived the project and acquired funding. S.L. developed and implemented the algorithm. S.L., T.Z., H.Z., and Z.Z. were responsible for methodology development. S.L., T.Z., and H.Z. validated the methods. S.L. and T.Z. wrote the original manuscript, with S.L. handling review and editing. R.Z., H.S., and Z.W. provided support in software development. All authors read and approved the final paper.

## Supporting information



Supporting Information

## Data Availability

The data that support the findings of this study are available from the corresponding author upon reasonable request.
