# Supplementary material for: SGCD: High‐Resolution Spatial Domain Characterization via Data Interpolation and Cell‐Type Deconvolution
Source: Adv Sci (Weinh). 2025 Jun 20;12(34):e06176. doi: 10.1002/advs.202506176 (PMC12442644; doi:10.1002/advs.202506176)
Supplement: Supplementary file 1 — Supporting Information [file ADVS-12-e06176-s001.pdf]

ADVANCED SCIENCE
Open Access

Supporting Information

for *Adv. Sci.*, DOI 10.1002/advs.202506176

SGCD: High-Resolution Spatial Domain Characterization via Data Interpolation and Cell-Type Deconvolution

*Tianjiao Zhang, Shenghe Li, Ruolan Zhang, Hongfei Zhang, Zhongqian Zhao, Hao Sun, Zhenao Wu and Guohua Wang**

Supplementary Figures

Figure S1. Manual annotation and comparison of spatial domains identified by SGCD, STAIG, Mucost, GraphST, STAGATE, and SpaGCN across 12 slices of the DLPFC dataset.

Figure S2. SGCD and other five benchmark methods for clustering results of human breast cancer tissue slices.

Figure S3. Results of T-tests conducted on SGCD, STAIG, Mucost, GraphST, STAGATE, and SpaGCN across 12 slices of the DLPFC dataset and the PDAC-B slice.

Figure S4. Application of SGCD to the PDAC-B slice.(A) Clustering results on the PDAC-B dataset using SGCD, STAIG, Mucost, GraphST, STAGATE, and SpaGCN.(B) Spatial distribution of cell types on the PDAC-B dataset, integrated with single-cell reference labels.

Figure S5. Boxplot of the results of SGCD ablation experiments on the human breast cancer dataset.

Figure S6. Comparison of ARI and NMI performance of JSD scatter, cosine similarity and Pearson correlation coefficients on the DLPFC dataset.

Figure S7. Time and memory overhead of SGCD on different datasets.(A)Total Processing Time for Each Dataset.(B)Memory Usage for Each Dataset.

Supplementary Tables

Table S1. Description of Spatial Transcriptomic Datasets Used in the Study.

Table S2. Description of Single-Cell RNA Sequencing Datasets Used in the Study.

Table S3. Optimal Parameter Recommendation.

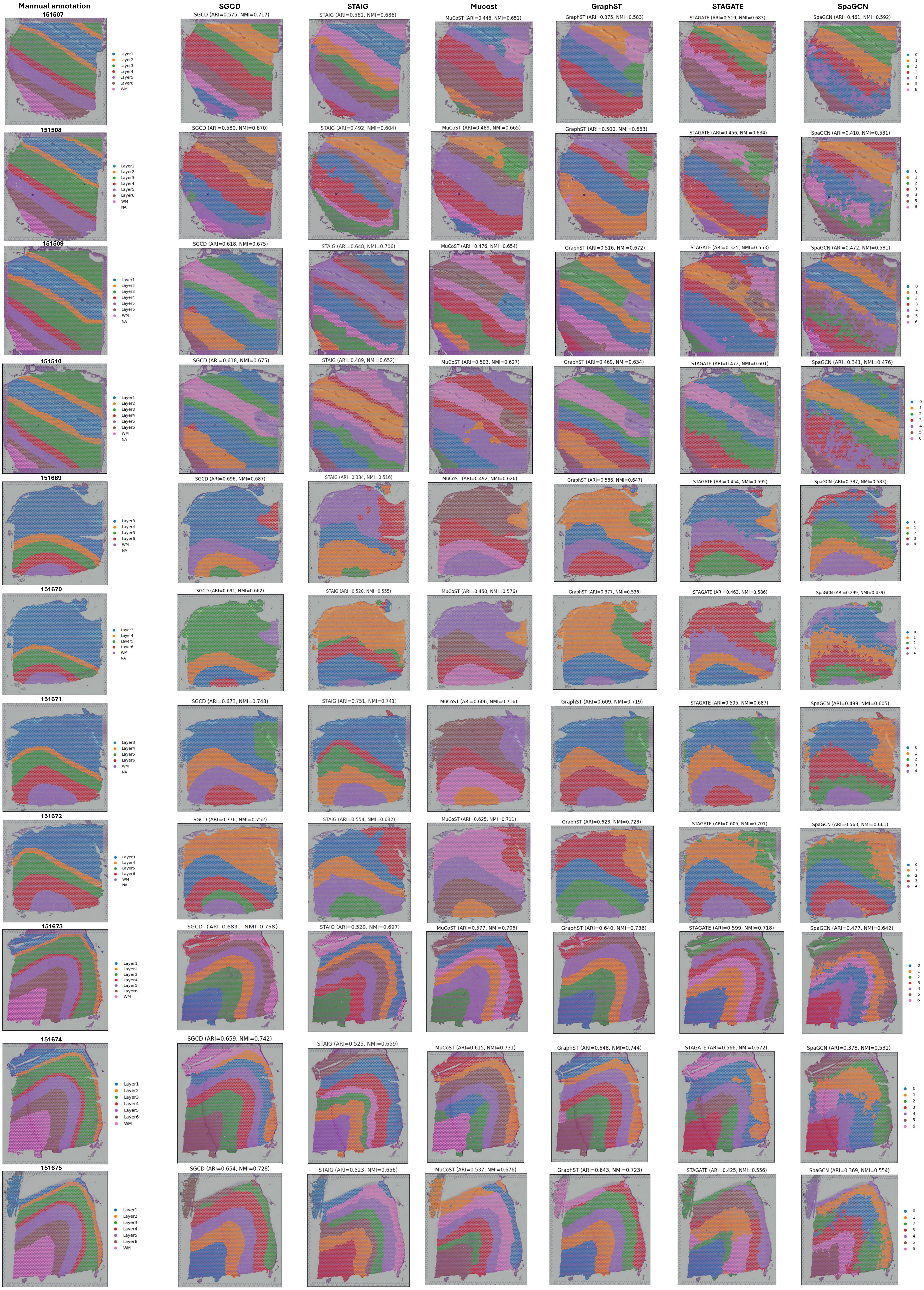
Mannual annotation
SGCD
STAIG
Mucost
GraphST
STAGATE
SpaGCN
151507
SGCD (ARI=0.575, NMI=0.717)
STAIG (ARI=0.561, NMI=0.686)
MuCoST (ARI=0.446, NMI=0.651)
GraphST (ARI=0.375, NMI=0.583)
STAGATE (ARI=0.519, NMI=0.683)
SpaGCN (ARI=0.461, NMI=0.592)
151508
SGCD (ARI=0.580, NMI=0.670)
STAIG (ARI=0.492, NMI=0.604)
MuCoST (ARI=0.489, NMI=0.665)
GraphST (ARI=0.500, NMI=0.663)
STAGATE (ARI=0.456, NMI=0.634)
SpaGCN (ARI=0.410, NMI=0.531)
151509
SGCD (ARI=0.618, NMI=0.675)
STAIG (ARI=0.648, NMI=0.706)
MuCoST (ARI=0.476, NMI=0.654)
GraphST (ARI=0.516, NMI=0.672)
STAGATE (ARI=0.325, NMI=0.553)
SpaGCN (ARI=0.472, NMI=0.581)
151510
SGCD (ARI=0.618, NMI=0.675)
STAIG (ARI=0.489, NMI=0.652)
MuCoST (ARI=0.503, NMI=0.627)
GraphST (ARI=0.469, NMI=0.634)
STAGATE (ARI=0.472, NMI=0.601)
SpaGCN (ARI=0.341, NMI=0.476)
151669
SGCD (ARI=0.696, NMI=0.687)
STAIG (ARI=0.334, NMI=0.516)
MuCoST (ARI=0.492, NMI=0.626)
GraphST (ARI=0.586, NMI=0.647)
STAGATE (ARI=0.454, NMI=0.595)
SpaGCN (ARI=0.387, NMI=0.583)
151670
SGCD (ARI=0.691, NMI=0.662)
STAIG (ARI=0.520, NMI=0.555)
MuCoST (ARI=0.450, NMI=0.576)
GraphST (ARI=0.377, NMI=0.536)
STAGATE (ARI=0.463, NMI=0.586)
SpaGCN (ARI=0.299, NMI=0.439)
151671
SGCD (ARI=0.673, NMI=0.748)
STAIG (ARI=0.751, NMI=0.741)
MuCoST (ARI=0.606, NMI=0.716)
GraphST (ARI=0.609, NMI=0.719)
STAGATE (ARI=0.595, NMI=0.687)
SpaGCN (ARI=0.499, NMI=0.605)
151672
SGCD (ARI=0.776, NMI=0.752)
STAIG (ARI=0.554, NMI=0.682)
MuCoST (ARI=0.625, NMI=0.711)
GraphST (ARI=0.623, NMI=0.723)
STAGATE (ARI=0.605, NMI=0.701)
SpaGCN (ARI=0.563, NMI=0.661)
151673
SGCD（ARI=0.683，NMI=0.758）
STAIG (ARI=0.529, NMI=0.697)
MuCoST (ARI=0.577, NMI=0.706)
GraphST (ARI=0.640, NMI=0.736)
STAGATE (ARI=0.599, NMI=0.718)
SpaGCN (ARI=0.477, NMI=0.642)
151674
SGCD (ARI=0.659, NMI=0.742)
STAIG (ARI=0.525, NMI=0.659)
MuCoST (ARI=0.615, NMI=0.731)
GraphST (ARI=0.648, NMI=0.744)
STAGATE (ARI=0.566, NMI=0.672)
SpaGCN (ARI=0.378, NMI=0.531)
151675
SGCD (ARI=0.654, NMI=0.728)
STAIG (ARI=0.523, NMI=0.656)
MuCoST (ARI=0.537, NMI=0.676)
GraphST (ARI=0.643, NMI=0.723)
STAGATE (ARI=0.425, NMI=0.556)
SpaGCN (ARI=0.369, NMI=0.554)
Layer1
Layer2
Layer3
Layer4
Layer5
Layer6
WM
NA

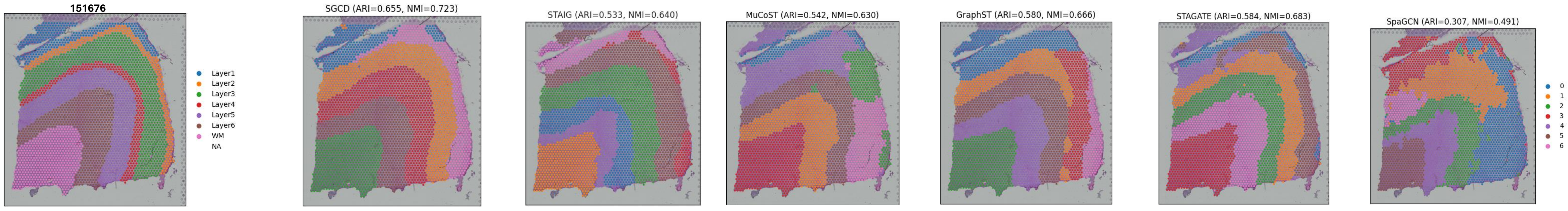

Figure S1. Manual annotation and comparison of spatial domains identified by SGCD, STAIG, Mucost, GraphST, STAGATE, and SpaGCN across 12 slices of the DLPFC dataset.

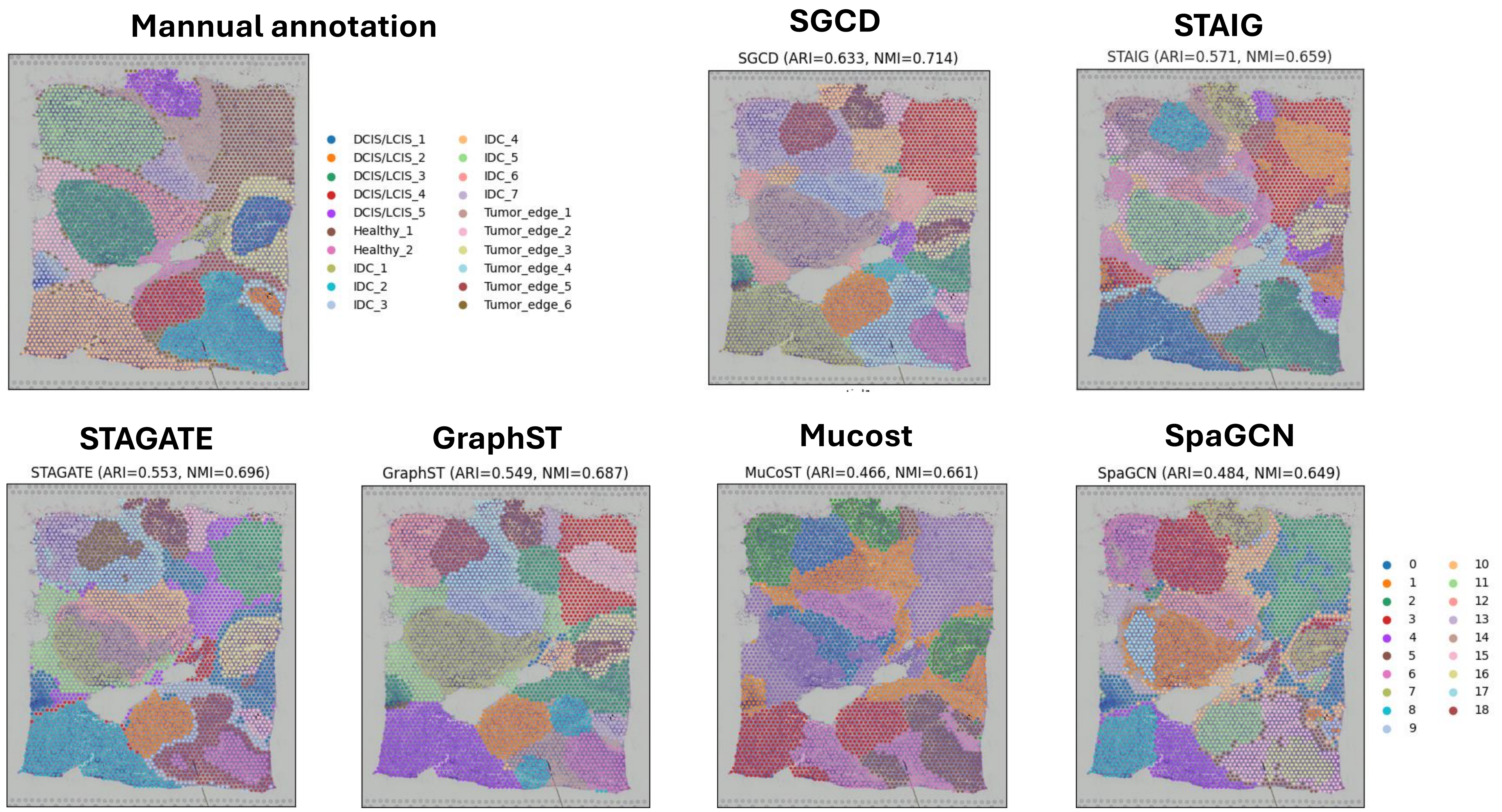

Figure S2. SGCD and other five benchmark methods for clustering results of human breast cancer tissue slices.

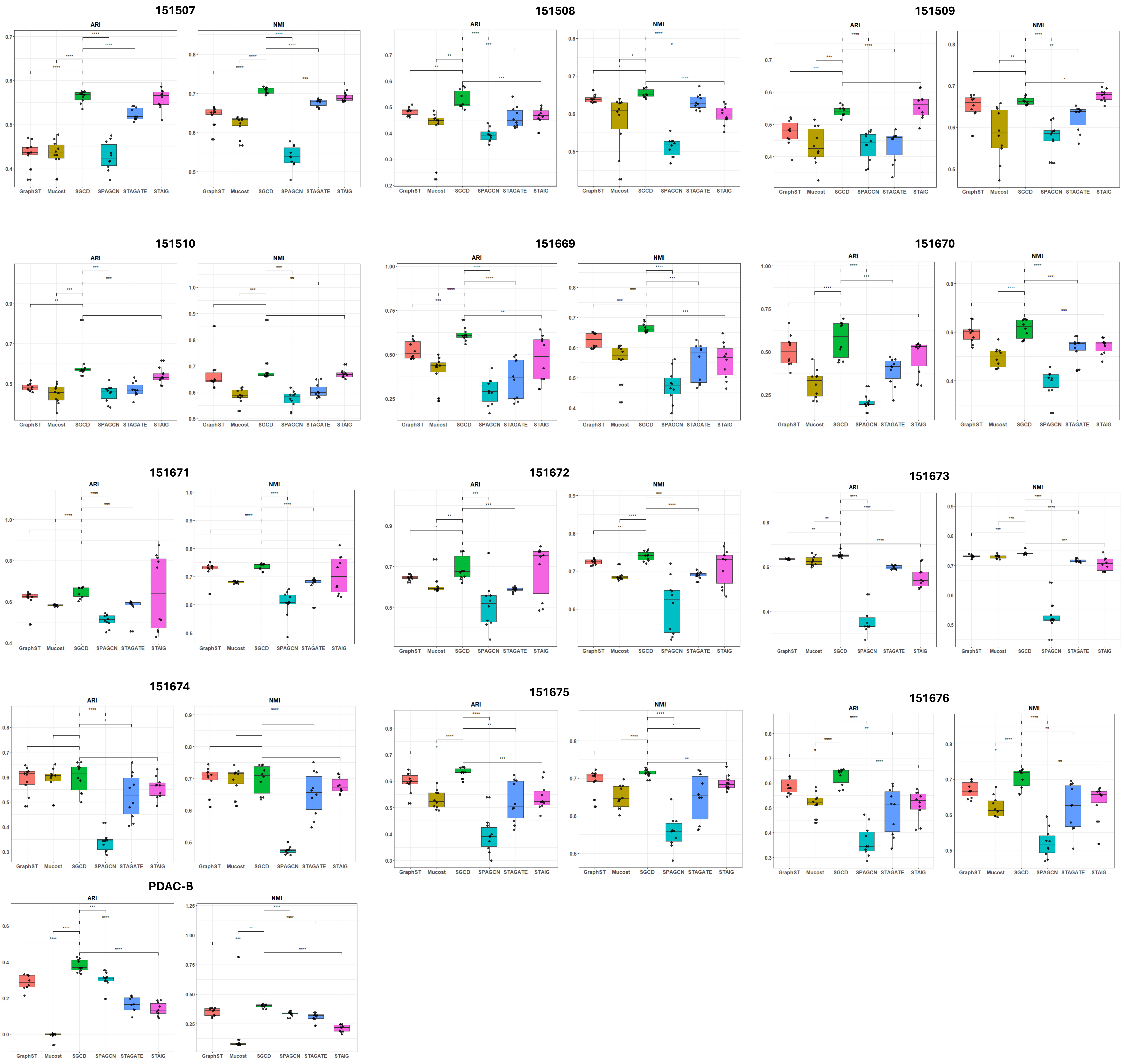

Figure S3. Results of T-tests conducted on SGCD, STAIG, Mucost, GraphST, STAGATE, and SpaGCN across 12 slices of the DLPFC dataset and the PDAC-B slice.

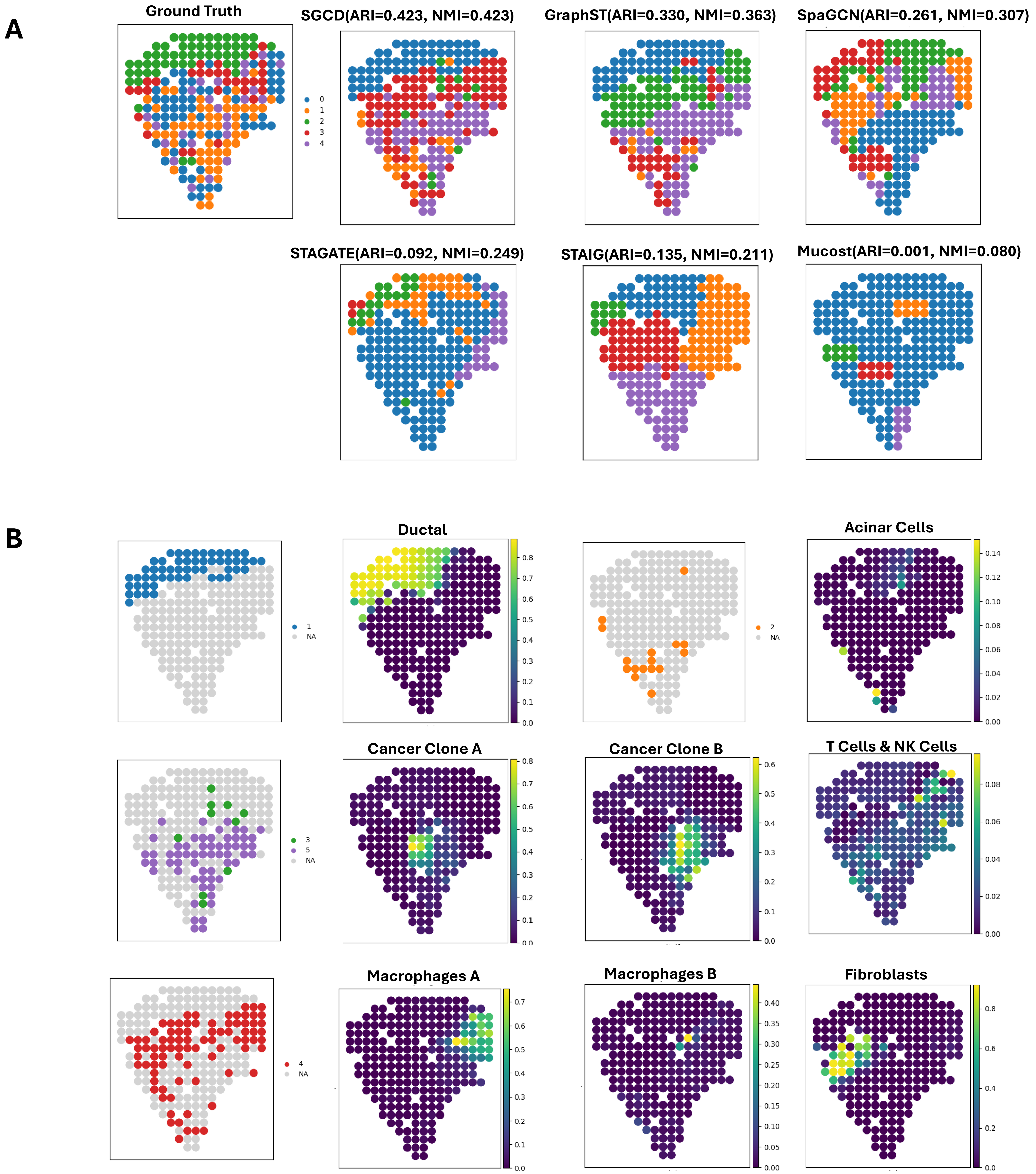

Figure S4. Application of SGCD to the PDAC-B slice.(A) Clustering results on the PDAC-B dataset using SGCD, STAIG, Mucost, GraphST, STAGATE, and SpaGCN.(B) Spatial distribution of cell types on the PDAC-B dataset, integrated with single-cell reference labels.

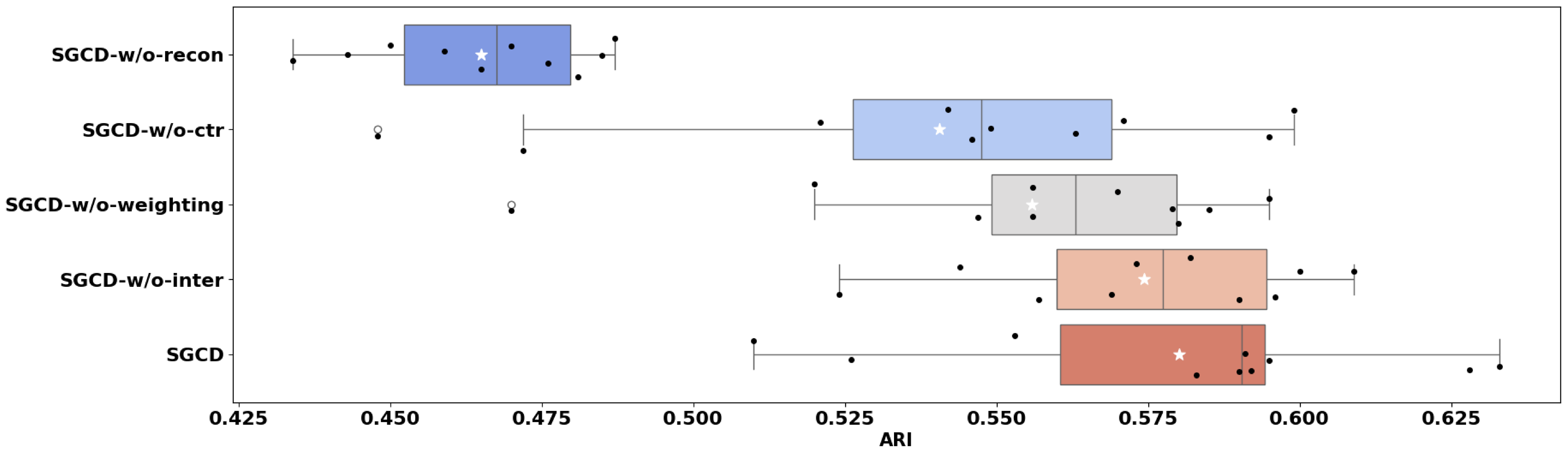

Figure S5. Boxplot of the results of SGCD ablation experiments on the human breast cancer dataset.

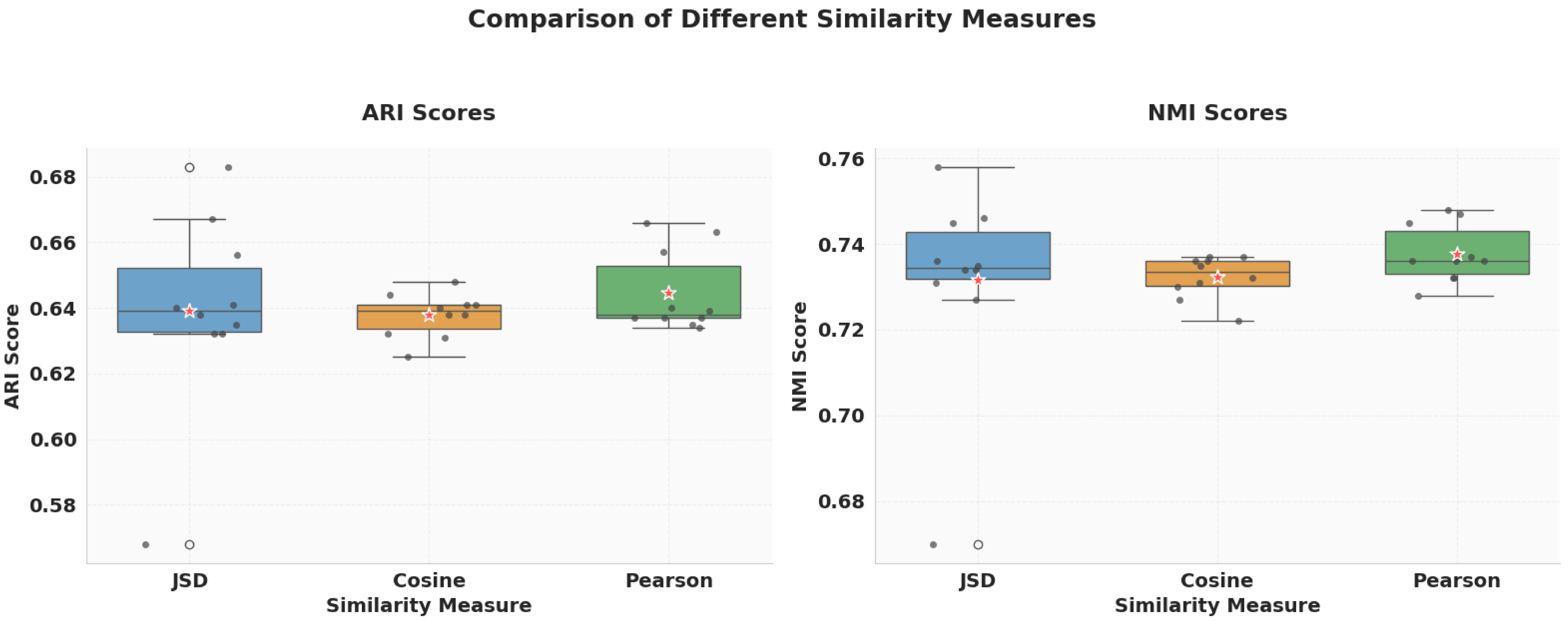

Figure S6. Comparison of ARI and NMI performance of JSD scatter, cosine similarity and Pearson correlation coefficients on the DLPFC dataset.

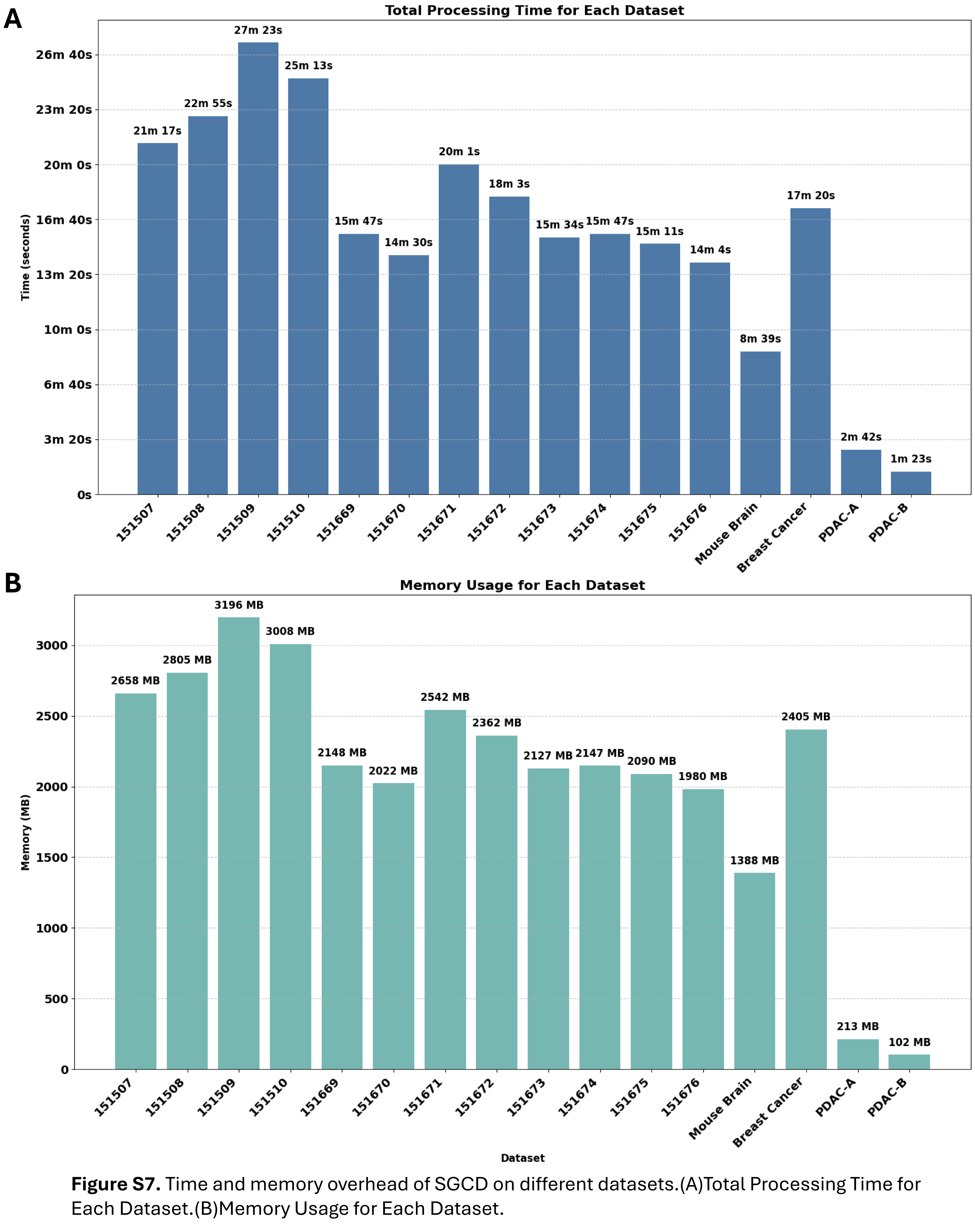

Figure S7. Time and memory overhead of SGCD on different datasets.(A)Total Processing Time for Each Dataset.(B)Memory Usage for Each Dataset.

Dataset Description

All datasets used in this study are publicly available and can be freely downloaded. We used spatial transcriptomic datasets from four different tissues (Table S1) and single-cell RNA sequencing datasets (Table S2).

The first dataset in Table S1 consists of 12 tissue slices from the human dorsolateral prefrontal cortex (DLPFC), collected using 10X Visium (http://research.libd.org/spatialLIBD/). Specifically, the DLPFC dataset is derived from 3 experimental subjects, with the number of spots per section ranging from 3,498 to 4,789, capturing a total of 33,538 genes. Each section has been manually annotated into 5 to 7 regions, including the DLPFC layers and white matter. Correspondingly, the first dataset in Table S2 is a single-cell RNA sequencing dataset, which includes high-throughput snRNA-seq of postmortem dorsolateral prefrontal cortex (BA9) tissue from MDD (major depressive disorder) subjects and controls using the 10X Genomics Chromium platform. This dataset includes 50,900 cells, 30,064 genes, and 6 cell types. The dataset is available on the GEO website (https://www.ncbi.nlm.nih.gov/geo/).

The second dataset is a mouse brain tissue dataset downloaded from the publicly available 10X Genomics resource (https://cf.10xgenomics.com/sample/spatial-exp/1.1.0/V1_Mouse_Brain_Sagittal_Anterior/). This dataset includes two parts, and we selected the anterior sagittal section. The selected section contains 2,695 spots, capturing 21,334 genes, and includes manual annotations of 52 regions by Long et al.[25]. Correspondingly, the second dataset in Table S2 is a single-cell RNA sequencing dataset derived from multiple cortical areas and the hippocampus of ~8-week-old male and female mice. It contains 74,973 cells, 45,769 genes, classified into 44 cell types. This dataset is available on the Brain Atlas website (https://portal.brain-map.org/atlases-and-data/rnaseq/mouse-whole-cortex-and-hippocampus-smart-seq).

The third dataset is from human breast cancer tissue samples obtained from malignant breast tissue. The selected section contains 3,798 spots, capturing 36,601 genes. This dataset is available on the 10X Genomics website (https://www.10xgenomics.com/resources/datas). Correspondingly, the third dataset in Table S2 is a single-cell RNA sequencing dataset that includes 549 primary breast cancer cells and lymph node metastases from 11 different patients. It includes 100,064 cells, 21,860 genes, classified into 9 cell types. This dataset is available on the GEO website (https://www.ncbi.nlm.nih.gov/geo/).

The fourth dataset is from human pancreatic ductal adenocarcinoma (PDAC) tissue sections from untreated PDAC patients. There are two tissue slices, PDAC-A and PDAC-B. The PDAC-A slice contains 428 spots and 19,738 genes, while the PDAC-B slice contains 224 spots and 19,738 genes. This dataset is available on the GEO website (https://www.ncbi.nlm.nih.gov/geo/). Referencing the SDMBench database, the two slices are annotated into 5 regions. Correspondingly, the fourth dataset in Table S2 is a single-cell RNA sequencing dataset derived from primary pancreatic cancer tissues of 6 patients. It includes 1,927 cells, 19,738 genes, classified into 17 cell types. This dataset is also available on the GEO website (https://www.ncbi.nlm.nih.gov/geo/).

Table S1. Description of Spatial Transcriptomic Datasets Used in the Study.

Dataset	Spots	Genes	Domains	Protocol	Species
**DLPFC_151507**	4221	33,538	7	10X Visium	Homo sapiens
**DLPFC_151508**	4381	33,538	7	10X Visium	H. sapiens
**DLPFC_151509**	4788	33,538	7	10X Visium	H. sapiens
**DLPFC_151510**	4595	33,538	7	10X Visium	H. sapiens
**DLPFC_151669**	3636	33,538	5	10X Visium	H. sapiens
**DLPFC_151670**	3484	33,538	5	10X Visium	H. sapiens
**DLPFC_151671**	4093	33,538	5	10X Visium	H. sapiens
**DLPFC_151672**	3888	33,538	5	10X Visium	H. sapiens
**DLPFC_151673**	3611	33,538	7	10X Visium	H. sapiens
**DLPFC_151674**	3635	33,538	7	10X Visium	H. sapiens
**DLPFC_151675**	3566	33,538	7	10X Visium	H. sapiens
**DLPFC_151676**	3431	33,538	7	10X Visium	H. sapiens
**Brain (Sagittal-Anterior)**	2695	32,285	52	10X Visium	Mus musculus
**Breast Cancer**	3,798	36,601	20	10X Visium	H. sapiens
**PDAC-A**	428	19,738	5	10X Visium	H. sapiens
**PDAC-B**	224	19,738	5	10X Visium	H. sapiens

Table S2. Description of Single-Cell RNA Sequencing Datasets Used in the Study.

Dataset	Cells	Genes	Cell Types	Species
**Single-nucleus RNA-seq in the post-mortem brain in major depressive disorder**	50,900	30,064	6	Homo sapiens
**Mouse Whole Cortex and Hippocampus SMART-seq**	74,973	45,769	44	Mus musculus
**Single cell RNA sequencing of primary breast cancer**	100,064	21,860	9	Homo sapiens
**single-cell RNA-seq in pancreatic ductal adenocarcinomas**	1927	19,738	17	Homo sapiens

Table S3. Optimal Parameter Recommendation.

Tissue	Section	*K*	*γ*
DLPFC	151507	4	0.8
	151508	1	0.9
	151509	4	0.9
	151510	1	0.9
	151669	10	0.7
	151670	10	0.8
	151671	1	0.4
	151672	1	0.4
	151673	4	0.2
	151674	3	0.1
	151675	6	0.5
	151676	2	0.6
Mouse brain	Section 1 (Sagittal-Anterior)	3	0.5
Breast Cancer	-	7	0.5
PDAC	A	3	0.5
	B	3	0.5
